# Proteome Profiling of the Dystrophic *mdx* Mice Diaphragm

**DOI:** 10.3390/biom13111648

**Published:** 2023-11-13

**Authors:** Olga Mucha, Małgorzata Myszka, Paulina Podkalicka, Bianka Świderska, Agata Malinowska, Józef Dulak, Agnieszka Łoboda

**Affiliations:** 1 Department of Medical Biotechnology, Faculty of Biochemistry, Biophysics and Biotechnology, Jagiellonian University in Krakow, Gronostajowa 7 Street, 30-387 Kraków, Poland; olgamuchaa@gmail.com (O.M.); malgorzata.myszka@doctoral.uj.edu.pl (M.M.); paulina.podkal@gmail.com (P.P.); jozef.dulak@uj.edu.pl (J.D.); 2 Doctoral School of Exact and Natural Sciences, Łojasiewicza 11 Street, 30-348 Kraków, Poland; 3 Mass Spectrometry Laboratory, Institute of Biochemistry and Biophysics, Polish Academy of Sciences, Pawińskiego 5a Street, 02-106 Warsaw, Poland; bianka@mslab-ibb.pl (B.Ś.); esme@ibb.waw.pl (A.M.)

**Keywords:** aquaporin 4, Duchenne muscular dystrophy, hydrogen sulfide, *mdx* diaphragm, CTH, CBS, MPST, osteopontin, periostin, proteome

## Abstract

*Mdx* mice with a spontaneous mutation in exon 23 of the *Dmd* gene represent the most common model to investigate the pathophysiology of Duchenne muscular dystrophy (DMD). The disease, caused by the lack of functional dystrophin, is characterized by irreversible impairment of muscle functions, with the diaphragm affected earlier and more severely than other skeletal muscles. We applied a label-free (LF) method and the more thorough tandem mass tag (TMT)-based method to analyze differentially expressed proteins in the diaphragm of 6-week-old *mdx* mice. The comparison of both methods revealed 88 commonly changed proteins. A more in-depth analysis of the TMT-based method showed 953 significantly changed proteins, with 867 increased and 86 decreased in dystrophic animals (*q*-value < 0.05, fold-change threshold: 1.5). Consequently, several dysregulated processes were demonstrated, including the immune response, fibrosis, translation, and programmed cell death. Interestingly, in the dystrophic diaphragm, we found a significant decrease in the expression of enzymes generating hydrogen sulfide (H_2_S), suggesting that alterations in the metabolism of this gaseous mediator could modulate DMD progression, which could be a potential target for pharmacological intervention.

## 1. Introduction

Duchenne muscular dystrophy (DMD) is a rare genetic disease related to the X chromosome, affecting 1 in 5000 to 6000 boys. This progressive and devastating disorder is caused by mutations in one of the largest genes in the human genome, *DMD*, which encodes dystrophin, a structural muscle protein [1,2,3]. Due to the two polyadenylation sites and seven tissue-specific independent promoters found in the *DMD* gene, as well as alternative mRNA splicing, several isoforms of dystrophin have been identified, including full-length proteins (427 kDa) and shorter versions like Dp260, Dp140, Dp116, Dp71, and Dp40 that have tissue-specific distribution [4].

In patients, the first symptoms of the disease are noticeable at the age of 1 to 3 years and manifest, among others, as a struggle to sit down, stand up, and walk. Between the ages of 8 and 14, diseased boys lose ambulation due to the gradual weakening of the muscles and become dependent on a wheelchair. The number of consequences associated with the lack of dystrophin in skeletal muscle is evident, mainly including a severe impairment of muscle function, dysregulation of the mechanical stability of muscle fibers during contraction, and impairment of numerous signaling pathways. These lead to myofiber necrosis and membrane leakage while activating the regenerative machinery. Consequently, constant cycles of damage and regeneration result in chronic inflammation with unbalanced polarization of M1–M2 macrophages, increased oxidative stress, and compromised myogenesis. Over the years, this causes the accumulation of fibrous and fatty tissues. Excess fat and connective tissues replace damaged myofibers, severely affecting muscle function. In addition to degeneration, inflammation, fibrosis, and muscle fiber regeneration, dysregulation of angiogenesis, impaired autophagy, or mitochondrial complications contribute to disease progression (reviewed in [5,6,7]).

The disease is invariably fatal, with death typically occurring from cardiorespiratory failure, predominantly cardiomyopathy [8,9,10,11]. Unfortunately, despite many years of intensive research, there is still no effective life-saving treatment for DMD. Glucocorticoids, despite the appearance of side effects, still serve as the gold-standard treatment for patients with DMD [5]. It is worth mentioning that the first gene therapy (Elevidys; Sarepta Therapeutics, Inc., Cambridge, MA, USA), which results in the production of micro-dystrophin, a shortened protein, has been recently accepted under accelerated approval by the FDA (22 June 2023). As there is an ongoing need to discover novel approaches that could, at least, lessen the severity of the disease, numerous research efforts have concentrated on identifying dysregulated genes and proteins. Such experiments are performed mostly in mouse models of the disease such as, for example, *mdx* mice, which develop DMD symptoms as a result of a spontaneous, nonsense point mutation (C-to-T transition) in exon 23 of the *Dmd* gene that leads to the loss of the major muscle dystrophin isoform Dp427 [12,13]. In contrast, in DMD patients, more than 7000 different mutations have been detected, with deletions of one or more exons being the most frequent (about 60–70% of all DMD cases) and point mutations and exonic duplication constituting about 26% and 10–15% of cases, respectively. Additionally, missense mutations, splice mutations, and subexonic insertions or deletions may result in the absence of dystrophin [14,15]. Such diversity in mutation types and high mutation rates hamper the application and success of gene therapies for all DMD patients.

Compared with humans, the course of the disease in dystrophic *mdx* mice is much milder. During the first 2 weeks of age, muscles of the *mdx* mouse cannot be distinguished from those of the healthy animal. Between 3 and 6 weeks, the first cycles of degeneration and regeneration occur [16]. Chronic inflammation, as evidenced by the infiltration of inflammatory cells, is also present in the skeletal muscles of *mdx* mice [17]. At 8 weeks of age, 80% of the fibers are centrally nucleated and their size is heterogeneous [18]. Other pathological changes observed in DMD mouse muscles are less severe, with only partial fibrosis and late-life fat accumulation suggesting a reduced regenerative decline in *mdx* mice compared with DMD patients (except in the diaphragm). Of note, several studies have demonstrated that not all dystrophic *mdx* muscles are equally vulnerable to muscular degeneration. Hind limb muscles of *mdx* mice undergo successful regeneration, while the *mdx* diaphragm exhibits a degenerative pattern similar to DMD patients’ skeletal muscles [19]. Early dystrophic alterations in the *mdx* diaphragm are already visible at 6 weeks of age [20], and its progressive degeneration, fibrosis, and inflammation persist throughout life [19]. This leads to hypoventilation and respiratory impairment [21]. Why the diaphragm is the most pathological muscle in dystrophin-deficient *mdx* mice is not really known. The reduced activation of the regenerative response in the diaphragm may result from the overproduction of extracellular matrix components and prominent myofibrosis, which inhibits regeneration (references in [22]).

As the diaphragm is severely affected in the *mdx* model, in the present study, we focus on this particular muscle to better understand the basic processes involved in disease progression and to reveal proteins that could serve as novel therapeutic targets. We applied two global proteomic methods: label-free (LF) and tandem mass tag (TMT)-based analysis, and we identified a number of differentially expressed proteins between normal and dystrophic diaphragms. The use of the LF method served as a preliminary study, giving us an insight into the changes caused by the lack of dystrophin in the diaphragm. A more thorough TMT-based approach, with sample fractionation, led to the identification of a higher number of changed proteins as a consequence of dystrophin deficiency. Of particular interest could be the decreased abundance of the hydrogen sulfide (H_2_S)-generating enzyme cystathionine γ-lyase (CTH, CSE, or CGL). Together with other endogenous enzymes, cystathionine β-synthase (CBS), and 3-mercaptopyruvate sulfurtransferase (MPST or 3-MST), these constitute the main enzymatic sources for H_2_S biosynthesis, being involved in antioxidant, anti-inflammatory, antifibrotic, and anti-apoptotic effects [23]. Recently, we [24] and others [25,26] proposed H_2_S-releasing molecules as a therapy to alleviate muscle defects in *mdx* mice and in the *C. elegans* model of DMD [27].

In summary, we found the dysregulation of many signaling pathways in the diaphragms of young 6-week-old *mdx* mice, including H_2_S generation. Therefore, we postulate that a better understanding of the role of this gaseous mediator in DMD may lead to the development of effective therapeutic strategies.

## 2. Materials and Methods

### 2.1. Mouse Model

All experiments were carried out following national and European legislation and according to the ARRIVE guidelines. Wild-type (WT, C57BL/10ScSnJ) and *mdx* (C57BL/10ScSn-*Dmd^mdx^*/J) mice were purchased from Jackson Laboratory (Bar Harbor, ME, USA; stock nos. 000476 and 001801, respectively). The animals were kept under specific pathogen-free (SPF) conditions in humidity (around 55 ± 10%)- and temperature (approximately 23 °C)-controlled cages with 14 h/10 h light/dark cycles and food and water ad libitum. Six-week-old male mice (6 WT and 5 *mdx* mice) were used for the proteomic analysis. Confirmatory experiments (Western blot and gene expression studies) were performed using 4–6 mice/genotype.

### 2.2. Protein Isolation

For the proteomic analysis, total protein was isolated from the diaphragm muscle of 6-week-old male mice and frozen in liquid-nitrogen-cooled isopentane, followed by homogenization in lysis buffer (1% sodium dodecyl sulfate (SDS, Sigma-Aldrich, St. Louis, MO, USA) in HEPES buffer solution (Gibco, Dublin, Ireland)) using TissueLyser (Qiagen, Hilden, Germany). Subsequently, the samples were incubated for 30 min, centrifuged (14,000× *g*, 10 min, room temperature (RT)), and the supernatants were collected and stored at −80 °C.

### 2.3. Total Protein Measurement

Total protein concentration was measured in a 96-well plate using the bicinchoninic acid (BCA) method. A 100 µL mixture of BCA solution and Copper(II) sulfate solution (both from Sigma-Aldrich, St. Louis, MO, USA) in a 50:1 ratio was mixed with 2 µL of protein lysate and incubated for 25 min at 37 °C. Each sample was analyzed in duplicate. After this time, the absorbance was measured at 562 nm using a Tecan Infinite M200 microplate reader.

### 2.4. Protein Digestion

Peptide digestion for the label-free (LF) and tandem mass tag (TMT) experiments were performed independently from the same sample sets. The internal standard (IS) for the TMT analysis was prepared by combining equal amounts of all the samples. For the LF and TMT experiments, samples were prepared according to the FASP protocol, with minor modifications [19]. The digestion of the samples was performed in 100 mM ammonium bicarbonate buffer (ABC) or 100 mM triethylammonium bicarbonate buffer (TEAB; pH 8.5) for the LF or TMT analysis, respectively. An amount of 50 µg (LF experiments) or 80 µg (TMT method) of each sample was diluted to 100 µL with the appropriate buffer. The cysteine groups were reduced by a 1 h incubation with 20 mM TCEP at 60 °C. Proteins were transferred onto a Vivacon 30 kDa molecular-weight cut-off filter (Sartorius Stedim, Kostrzyn Wlkp, Poland). The samples were spun at 14,500× *g* for 30 min and washed with 100 µL urea solution (8 M urea in 100 mM ABC or TEAB buffer). Cysteines were alkylated by a 20 min incubation at RT with 40 mM iodoacetamide. The filters were washed three times with urea solution and three times with buffer, respectively. After each addition, the samples were centrifuged until the cut-off filter was dry. Digestion was performed overnight using a trypsin/Lys-C mix (Promega, Madison, WI, USA) in a 1:20 enzyme-to-protein ratio at 37 °C. Peptides were eluted from the spin filters by two additions of 50 µL buffer and one of 500 mM NaCl solution. The Speedvac-dried samples were resuspended in 100 µL of 100 mM TEAB buffer for TMT labeling or with 200 µL of 0.1% formic acid (FA) and 2% acetonitrile (ACN) in water for direct LF analysis.

### 2.5. TMT Labeling

The peptides were labeled with TMT 10plex (Thermo Fisher Scientific, Waltham, MA, USA) tags in 41 µL ACN for 1 h with vortexing. Internal standards were labeled with 131 TMT tags. The reaction was quenched by adding 8 µL of 5% hydroxylamine. The labeling efficiency was tested using MS parameters as described below in the Mass spectrometry and Data analysis sections, with TMT used as a variable modification. The combined TMT sets were dried, resuspended in water, and desalted using two 30 mg Oasis HLB columns (Waters, Milford, MA, USA). Briefly, the cartridges were preconditioned with 1 mL of methanol and washed with MS-grade water. After sample loading, columns were rinsed with 1 mL of water and the peptides were eluted with 400 µL of 75% ACN and 0.1% FA. The aliquots were dried and resuspended in 500 µL of 10 mM ammonium hydroxide.

### 2.6. Reversed-Phase Peptide Fractionation of TMT-Labeled Peptides

TMT-labeled peptides were fractionated using the high-pH reversed-phase method into 25 fractions. The system and gradient parameters are described in our previous publication [28].

### 2.7. Mass Spectrometry

An amount of 2 µg of unlabeled samples or 20 µL of each fraction from the TMT sets was analyzed using a liquid chromatography–tandem mass spectrometry (LC-MS) system composed of an ACQUITY UPLC M-Class System (Waters, Milford, MA, USA) coupled to a QExactive mass spectrometer (Thermo Fisher Scientific, Waltham, MA, USA) via a nanoFlex ion source. The peptides were loaded onto the C18 pre-column (180 µm × 20 mm, Waters, Milford, MA, USA) and separated using the nanoAcquity BEH C18 column (75 µm × 250 mm, 1.7 µm, Waters, Milford, MA, USA) in an ACN gradient (0–35% ACN in 0.1% FA, 160 min) at a flow rate of 250 nL/min. The acquisition was performed using a data-dependent method, with the top 12 precursors selected for MS2 analysis with a normalized collision energy (NCE) of 27. Full MS scans were acquired at a resolution of 70,000 with an AGC of 1 × 10^6^. For the LF analysis, the mass range was set to 300–1650 *m*/*z*, with a maximum injection time of 60 ms. During MS2 scans, the maximum injection time was 60 ms, and the AGC target was 2 × 10^5^, with a resolution of 17,500. The isolation window was 3.0 *m*/*z*, and the dynamic exclusion was set to 30 s. For the TMT analysis, the parameters were as follows: mass range, 300–1600 *m*/*z*; full MS maximum injection time, 120 ms; MS2 maximum injection time, 120 ms; AGC target, 5 × 10^5^; an MS2 resolution of 35,000; isolation window, 1.2 *m*/*z;* and a dynamic exclusion of 30 s.

### 2.8. Data Analysis

#### 2.8.1. LF Analysis

The acquired data were preprocessed with the Mascot Distiller software (v. 2.6, MatrixScience, London, UK) and searched with the Mascot Search Engine (MatrixScience, London, UK, Mascot Server 2.6) against the Swissprot-derived mouse protein database (version 2019_09; 17,032 sequences). To reduce mass errors, the peptide and fragment mass tolerance settings were established separately for individual raw data files after measured mass recalibration, as previously described [29]. The rest of the search parameters were as follows: enzyme, trypsin; missed cleavages, 1; fixed modifications, carbamidomethyl (C); variable modifications, oxidation (M); instrument, HCD. A statistical assessment of the confidence of peptide assignments was based on the target/decoy database search strategy [30]. This procedure provided *q*-value estimates for each peptide spectrum match in the dataset. All queries with *q*-values > 0.01 were removed from further analysis, as well as proteins with less than two peptides in the overall dataset and proteins identified by a subset of peptides from another protein. We conducted mass calibration and data filtering using the in-house MScan software (version 2.0.4), accessible at http://proteom.ibb.waw.pl/mscan/ (accessed on 18 October 2018). The lists of identified peptides were consolidated into a unified list. This list was overlaid onto 2-D heatmaps generated from LC-MS profile datasets by tagging the peptide-related isotopic envelopes with corresponding peptide sequence tags based on the measured/theoretical mass difference, elution time deviation, and the match between the theoretical and observed isotopic envelopes. A detailed description of our quantitative extraction procedure, implemented through in-house software, can be found in [31]. The abundance of each peptide was determined as the volume of a 2-D fit to the monoisotopic peak of the tagged isotopic envelope. Quantitative data, alongside peptide/protein identifications, were exported to text files for further statistical analysis using the Diffprot software (version 1.5.19) [29]. Diffprot was configured with the following parameters: the number of random peptide sets = 10^6^; clustering of peptide sets—only when 90% identical; normalization by LOWESS.

#### 2.8.2. TMT Analysis

Offline recalibration, as well as peptide and protein identification, was performed in the MaxQuant/Andromeda software suite (version 1.6.2.3) [32] using *Mus musculus* protein sequences derived from the Swissprot database (version 2019_05). The search included only tryptic peptides, with carbamidomethyl (C) set as a fixed modification and oxidation (M) and acetyl (protein N-term) as variable modifications. Quantification with TMT labels was specified to obtain reporter values for further analysis. A reverse database was used for the validation of target/decoy statistical results, and the false discovery rate (FDR) was set to 0.01. Protein groups, along with quantitative data, were further analyzed in Perseus (version 1.6.0.6) [33]. Data were cleaned (hits only identified by site, originating from a reversed database, and contaminants were removed). The TMT reporter values were normalized by internal reference scaling (IRS [34]) using reference channels in order to take into account the fact that the data were distributed over two separate TMT experiments. Data were then log-transformed for better distribution, and missing values were replaced with data from a normal distribution (width 0.3, downshift 1.8) separately for each column. Subsequently, a two-sample *t*-test with permutation-based FDR was performed to compare expression changes between groups. The significance threshold for the resulting *q*-value was 0.05. Data were analyzed using the Ingenuity Pathway Analysis (IPA) (Qiagen, Hidden, Germany, https://www.QIAGENbioinformatics.com/products/ingenuitypathway-analysis, accessed on 18 October 2018) [35] and the Search Tool for the Retrieval of Interacting Genes/Proteins (STRING) database for known and predicted interactions (default settings were chosen for the analysis: network type: full STRING network; required score: medium confidence (0.400); FDR stringency: medium (5 percent)) [36].

### 2.9. Western Blot

Muscles (diaphragms) were isolated, snap-frozen in liquid nitrogen, and subsequently stored at −80 °C for further analyses. For tissue homogenization, a TissueLyser (Qiagen, Hilden, Germany) was used. The tissue homogenates were suspended in ice-cold lysis buffer (PBS containing proteinase inhibitors (Roche Diagnostics, Mannheim, Germany) and 1% Triton X100 (BioShop, Burlington, ON, Canada). The total protein concentration was measured using the BCA (Sigma-Aldrich, St. Louis, MO, USA) method. For the Western blot, 20 μg of protein was used, and the protein lysate was processed as previously described [37]. For protein detection, the following primary antibodies were used: rabbit polyclonal anti–CTH (Abcam, Cambridge, UK; ab151769, 1:1000), rabbit polyclonal anti-MPST (Sigma-Aldrich, St. Louis, MO, USA; HPA001240, 1:500), rabbit polyclonal anti-CBS (Proteintech, Manchester, UK; 14787-1-AP, 1:1000), mouse monoclonal anti-tubulin (Sigma-Aldrich, St. Louis, MO, USA; T9026, 1:500); mouse monoclonal anti-vinculin (Sigma-Aldrich, St. Louis, MO, USA; V9131, 1:200) rabbit polyclonal anti-aquaporin-4 (Novus Biologicals, Centennial, CO, USA; NBP2-92886, 1:1000). The following secondary antibodies (conjugated with HRP) were used: anti-rabbit IgG (Cell Signaling Technology; 7074, 1:10,000) for the detection of CTH, MPST, and aquaporin-4 and anti-mouse IgG (BD Biosciences, San Jose, CA, USA; 554002, 1:5000) for the detection of vinculin and tubulin.

### 2.10. Enzyme-Linked Immunosorbent Assay (ELISA)

To obtain protein lysates, diaphragm fragments were homogenized in 1% Triton X-100 in PBS using TissueLyser (Qiagen, Hilden, Germany) and then centrifuged at 10,000× *g* for 10 min at 4 °C. The BCA assay (Sigma-Aldrich, St. Louis, MO, USA) was used to determine the total protein concentration. The level of osteopontin (OPN) was analyzed in 100 µg of protein lysate following the manufacturer’s protocol (R&D Systems, Minneapolis, MN, Canada). The results are presented as pg/mg protein.

### 2.11. Histological Analysis

Directly after collection, muscles were pre-treated for a few minutes at an optimal cutting-temperature (OCT) medium (Leica, Wetzlar, Germany). Then, they were transferred to fresh OCT-containing tubes, frozen in liquid-nitrogen-cooled isopentane, and stored at −80 °C. Sections of 10 µm thickness were cut on a cryotome (Leica, Wetzlar, Germany) and directly put onto poly-L-lysine-coated slides. After ~2 h air-drying at RT, the sections were stored at −20 °C for further analyses. Before staining, the frozen sections were removed from −20 °C, left at RT for ~45 min, and fixed with 4% paraformaldehyde (PFA) (Sigma-Aldrich, St. Louis, MO, USA). For hematoxylin and eosin (H&E) staining, tissue sections were immersed in hematoxylin solution (Sigma-Aldrich, St. Louis, MO, USA) for 12 min, washed with running tap water (15 min), and stained for 1.5 min in 0.1% eosin solution (Sigma-Aldrich, St. Louis, MO, USA). Sections were also stained with a Trichrome Stain (Masson) Kit (Sigma-Aldrich, St. Louis, MO, USA) according to the manufacturer’s protocol. After H&E and Masson’s trichrome staining, the sections were dehydrated by increasing concentrations of ethanol (70%, 96% (×2), 99.8% (×2) of aqueous EtOH (Avantor Performance Materials Poland S.A., Gliwice, Poland), then cleared in xylene (Sigma-Aldrich, St. Louis, MO, USA) and sealed in Histofluid medium (Chemilab, Tarnobrzeg, Poland). Pictures of the slides were taken using a Nikon Eclipse Ti microscope (Nikon, Tokyo, Japan).

### 2.12. RNA Isolation, Reverse Transcription, and Quantitative Real-Time PCR (qRT-PCR)

Total RNA from the diaphragm was isolated using the Chomczynski–Sacchi method [38]. Before this isolation, diaphragm fragments were protected in RNAlater Stabilization Solution (Millipore Sigma/Sigma-Aldrich, St. Louis, MO, USA), snap-frozen in liquid nitrogen, and stored at −80 °C. Briefly, diaphragm lysis was performed using QIAzol Total RNA Isolation Reagent (Qiagen, Hilden, Germany), followed by chloroform extraction and isopropanol precipitation. The concentration and quality of the RNA obtained were determined by measuring the 260/280 nm and 260/230 nm ratios using a NanoDrop Spectrophotometer (Thermo Fisher Scientific, Waltham, MA, USA). Reverse transcription was performed with recombinant M-MuLV reverse transcriptase (Thermo Fisher Scientific, Waltham, MA, USA) as previously described [37]. A mixture containing cDNA, SYBR Green PCR Master Mix (SYBR Green qPCR Kit), and specific primers that recognize the murine genes indicated in Table 1 was prepared, and the qRT-PCR was performed using StepOne Plus Real-Time PCR (Applied Biosystems, Thermo Fisher Scientific, Waltham, MA, USA). The *Eef2* gene, which is stably expressed in WT and *mdx* diaphragms, was used as a housekeeping gene, and the comparative cycle threshold (C_t_) method was utilized for the relative quantification of gene expression, calculated as a log_2_FC*_mdx_*__vs_WT_ based on the published protocol [39].

### 2.13. Statistical Analysis

Statistical analysis was performed by the unpaired 2-tailed Student’s *t*-test and Grubbs’ outlier test. Data from the qRT-PCR, ELISA, and densitometric analysis of Western blots are presented as mean ± SEM, where *p* < 0.05 indicates statistical significance. The exact *n* number of animals per group is included in the figure legend.

## 3. Results

### 3.1. Preliminary Comparative Results from Both the Label-Free (LF) Method and the Tandem Mass Tag (TMT) Approach

To better understand the pathology of DMD, we performed a proteomic analysis of diaphragm muscles harvested from 6-week-old WT and *mdx* animals. This gave us insight into the molecular pathways affected by dystrophin depletion. We used two methodological approaches: LF as a preliminary study and a label-based TMT strategy for a more detailed analysis. In the LF method, samples without fractionation were handled and individually examined. For the TMT analysis, chemical labeling was utilized at the peptide level. Once labeled, the samples were mixed, fractionated, and analyzed simultaneously.

**Figure 1:**
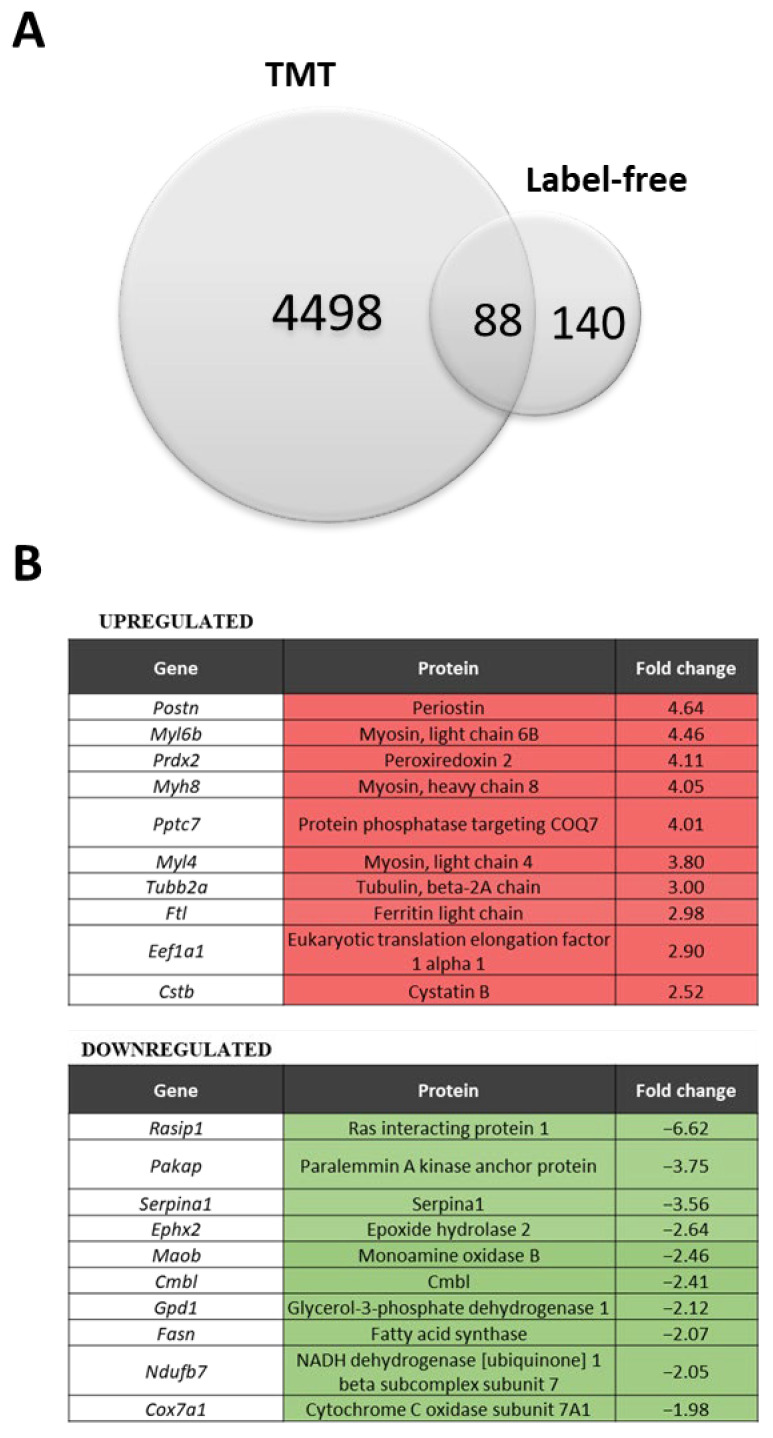

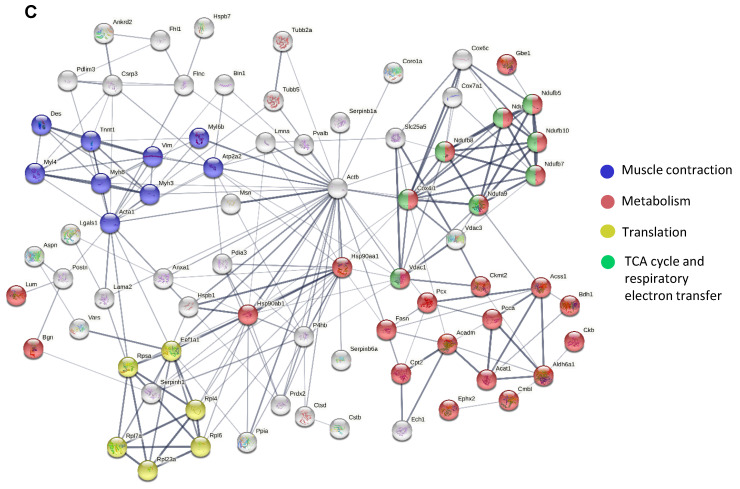
Comparison of the label-free (LF) and label-based isobaric tandem mass tag (TMT) analysis. (**A**) Venn diagram of differentially expressed proteins (dystrophic versus WT), showing the overlap between the TMT and LF methods. (**B**) List of 10 highly increased and decreased proteins in the dystrophin-deficient *mdx* diaphragm detected by both methods; fold change from the LF method. (**C**) A map of potential protein interactions (proteins detected by both methods) generated with the STRING database (version 9.1) of known and predicted protein associations that include direct physical and indirect functional protein linkages. The thickness of the line reflects confidence in the protein–protein interaction. Examples of the enriched Reactome pathways (medium confidence (0.400); FDR stringency: medium (5 percent)) are marked with colors.

In the LF approach, we were able to identify 2204 proteins, of which 140 were differentially expressed (Supplementary Table S1), while in the TMT analysis, among the 7085 proteins detected, 4498 proteins were differentially expressed between WT and the dystrophic diaphragm (Supplementary Table S2) (*q*-value < 0.05) (Figure 1A). A total of 88 proteins were identified as significantly changed in both methods (Figure 1A). The LF method showed several proteins with increased and decreased abundance in the *mdx* diaphragm (Figure 1B). Basic analysis using the STRING database (and the incorporated Reactome database) [36] performed on the 88 shared proteins revealed several processes, including muscle contraction, metabolism, the citric acid (TCA) cycle, respiratory electron transfer, and translation, that were considerably enriched in our model (Figure 1C).

### 3.2. TMT Analysis Revealed Proteins with Increased Rather Than Decreased Abundance in Dystrophic Animals

**Figure 2:**
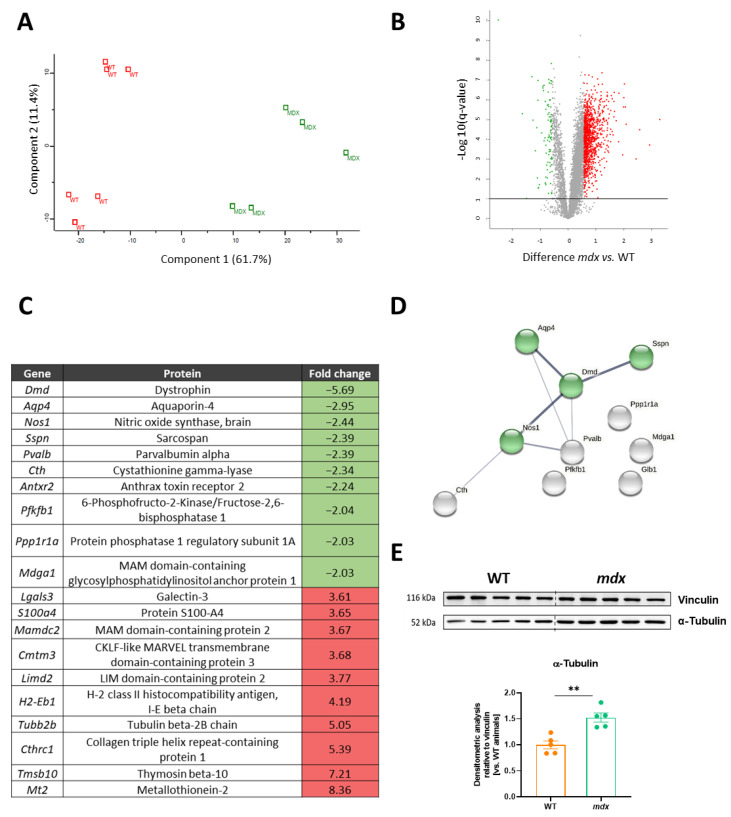
Differentially expressed proteins and pathway analysis in dystrophic muscle. Differentially expressed proteins obtained in the TMT analysis were visualized by (**A**) principal component analysis (PCA) and (**B**) volcano plot (showing the relationship between the log2 fold change (FC) and the level of significance (−log10(*q*-value)); green—decreased protein abundance; red—increased protein abundance; *q*-value 0.05, FC 1.5). (**C**) The list of the top 10 most significantly changed proteins in the dystrophic diaphragm. (**D**) Among the proteins with lower abundance, the connection between dystrophin (DMD), aquaporin-4 (AQP4), nitric oxide synthase-1 (NOS1), and sarcospan (SSPN) was found and visualized with the STRING database (version 9.1). (**E**) The tubulin level was higher in the dystrophic diaphragm, as confirmed by Western blot (Figure S1) and densitometric analysis (*n* = 5–6/group); results shown as mean ± SEM; ** *p* < 0.01.

In the following analysis, we decided to look more thoroughly at the results obtained using the TMT technique. For the protein group analysis along with data quantification, 6 WT and 5 *mdx* animals were taken into consideration. Principal component analysis (PCA) demonstrated a satisfactory clustering of samples within the same genotype and a substantial separation of dystrophic animals from their WT counterparts (Figure 2A). The results of the experimental group comparisons were also presented in the volcano plot (Figure 2B). To select and focus on the most prominently changed proteins, the following criteria were used: *q*-value = 0.05, and the threshold for the change was set at 1.5. As a result, we detected 953 significantly altered proteins: 867 of them were increased, while the remaining 86 proteins were decreased in *mdx* mice.

When analyzing the most changed proteins, we observed that among the 10 with the lowest abundance, we can distinguish a few interconnected proteins (Figure 2C,D). Among the 10 highly increased proteins, which included metallothionein 2, thymosin beta-10, collagen triple-helix-repeat-containing protein 1, and tubulin-beta 2 chain, we did not observe significant connections. In addition to the increased level of β-tubulin identified in the TMT analysis, the expression of the α-tubulin isoform was also higher in the *mdx* diaphragms, as shown by Western blot and its semi-quantification (Figure 2E).

### 3.3. Immune-System-Related Pathways Are Strongly Enriched in the Diaphragm Muscle of Dystrophic Animals

**Figure 3:**
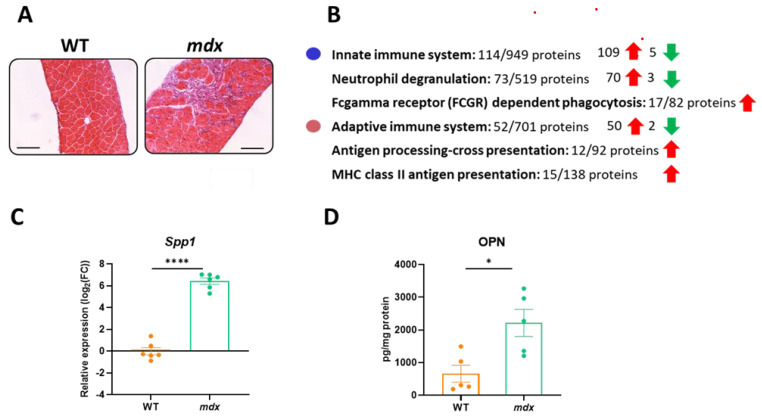
The increase in immune-system-related proteins in the diaphragm muscle of *mdx* mice. (**A**) Histological evaluation of inflammation based on hematoxylin and eosin (H&E) staining indicates increased inflammatory cell infiltration; scale bar: 100 μm. (**B**) The STRING analysis revealed changes in innate and adaptive immune responses. (**C**,**D**) The increased expression of *Spp1*/osteopontin revealed by STRING analysis was confirmed by quantitative real-time PCR and the ELISA test; *n* = 5–6/group; results shown as mean ± SEM; * *p* < 0.05, **** *p* < 0.0001.

Changes in the immune response are one of the hallmarks of DMD and increased infiltration of inflammatory cells in the dystrophic diaphragm is prominent, as shown by H&E staining (Figure 3A). When differentially expressed proteins identified in the TMT method were analyzed using the STRING database (and the incorporated Reactome database), we observed that both innate and adaptive immune responses were affected (mainly as an increased abundance of related proteins) (Figure 3B). Among the differentially expressed proteins, an increase in osteopontin (*Spp1*; OPN) level (fold change: 2.19 in the TMT analysis) was confirmed by qRT-PCR and ELISA, respectively (Figure 3C,D).

### 3.4. The Organization of the Extracellular Matrix (ECM) Is Disturbed in the Diaphragm of Dystrophic Mice

**Figure 4:**
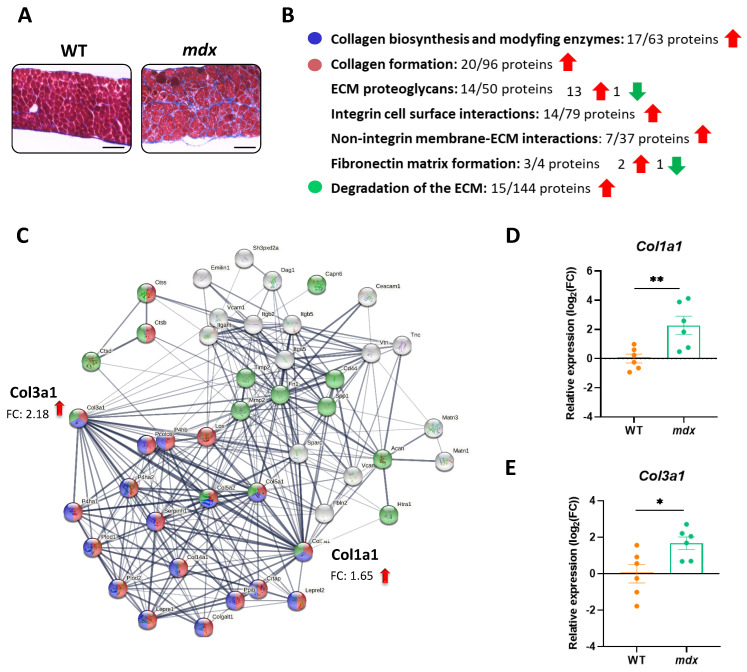
Increased fibrosis is a hallmark of dystrophic diaphragms. (**A**) Histological evaluation of Masson’s trichrome staining showed increased collagen deposition in the diaphragms of *mdx* mice; scale bar: 100 μm. (**B**,**C**) The STRING analysis revealed changes in the level of pro-fibrotic proteins, among others, that control collagen biosynthesis and extracellular matrix (ECM) organization. The thickness of the line reflects the confidence in the protein–protein interaction. Examples of the enriched (medium confidence (0.400); FDR stringency: medium (5 percent)) pathways are marked with colors. (**D**,**E**) Increased expression of *Col1a1* and *Col3a1* revealed by STRING analysis was confirmed by quantitative real-time PCR; *n* = 6; results are shown as mean ± SEM; * *p* < 0.05, ** *p* < 0.01.

The disorganization of the ECM, associated with fibrosis, is one of the most typical characteristics related to DMD. Compared with their WT counterparts, the accumulation of fibrotic tissue is detected in the diaphragms of dystrophic animals as shown by Masson’s trichrome staining (collagen is stained blue) (Figure 4A). Moreover, our proteomic results demonstrated that dystrophin deficiency leads to an increase in the abundance of several proteins related to pathways responsible for the proper formation and modification of the ECM, including, for example, collagen biosynthesis and modifying enzymes, collagen formation, and degradation of the ECM (Figure 4B). Within the affected proteins, we can find the high-abundance collagens 1 and 3 (encoded by *Col1a1* and *Col3a1*) (Figure 4C). Our observation was further strengthened by the estimation of the mRNA level of both proteins showing similar expression patterns (Figure 4D,E).

### 3.5. The Lack of Dystrophin Affects Protein Metabolism and Processes Related to the Cellular Response to Stress

**Figure 5:**
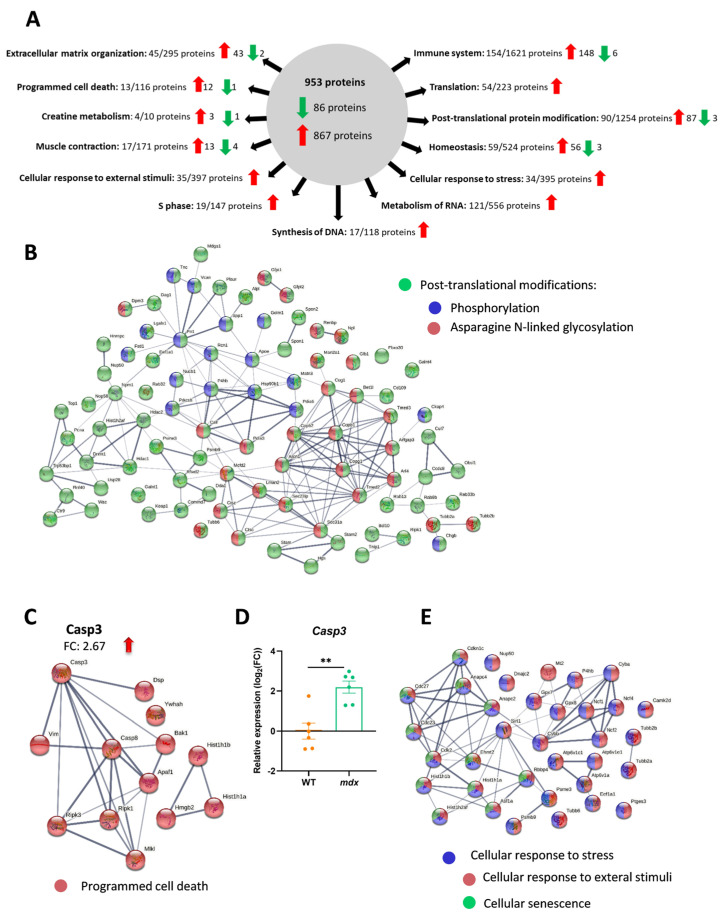
Complex changes in distinct molecular processes and pathways are revealed by proteomic analysis of the dystrophic diaphragm. (**A**) Among 953 differentially regulated proteins (q-value = 0.05, with the threshold for the fold change set at 1.5), basic analysis using the STRING database indicated several significantly enriched (medium confidence (0.400); FDR stringency: medium (5 percent)) molecular processes and pathways. (**B**) Post-translational-modification-related proteins are among those highly affected by dystrophin deficiency. (**C**) Programmed cell death exemplified by the high-abundance caspase-3 was evident and confirmed by (**D**) quantitative real-time PCR; *n* = 6; results are shown as mean ± SEM; ** *p* < 0.01. (**E**) Cellular response to stress was also strongly influenced in *mdx* mice. (**B**,**C**,**E**) The thickness of the line reflects the confidence in the protein–protein interaction. Examples of enriched pathways are marked with colors.

In addition to changes in inflammation and fibrosis, basic analysis using the STRING database (along with the incorporated Reactome and KEGG databases) revealed additional significantly enriched (FDR stringency: 5 percent) molecular processes and pathways that are crucial to proper muscle function, such as programmed cell death (13 out of 113 proteins; FDR: 0.0433), creatine metabolism (4 out of 10 proteins; FDR: 0.0388), muscle contraction (17 out of 171 proteins; FDR: 0.0357), cellular response to external stimuli (35 out of 397 proteins; FDR: 0.0026) and stress (34 out of 395 proteins; FDR: 0.0047), S phase (19 out of 147 proteins; FDR: 0.0015), cellular senescence (12 out of 124 proteins; FDR: 4.79 × 10^−16^) and DNA synthesis (17 out of 118 proteins; FDR: 0.0012), RNA metabolism (121 out of 556 proteins; FDR: 1.84 × 10^−41^), overall homeostasis (59 out of 524 proteins; FDR: 7.95 × 10^−9^), translation (54 out of 223 proteins; FDR: 4.46 × 10^−20^), and post-translational modification (90 out of 1254 proteins; FDR: 8.52 × 10^−5^) (Figure 5A; the full list of pathways is attached as Supplementary Table S3).

Among the processes significantly affected by the lack of dystrophin, post-translational modification, with 87 high-abundance proteins (only 3 with decreased levels), was noted. Among them, we were able to distinguish phosphorylation and asparagine N-linked glycosylation as processes potentially affected in the diaphragm of *mdx* mice (Figure 5B). In addition, programmed cell death was significantly enriched in dystrophic animals, with a 2.67-fold change in caspase 3 levels (Figure 5C). Interestingly, we also observed a similar pattern of change when the *Casp3* mRNA level was analyzed (Figure 5D). However, analysis of the cleaved (activated) form of the caspase 3 protein is warranted.

Furthermore, programmed cell death is one of the ways, opposite to the activation of pathways that promote survival, in which cells respond to stress. Our results also strongly suggest changes in various proteins involved in the cellular response to stressful conditions. Within this group, we can distinguish proteins related to the response to external stimuli and cellular senescence, among other pathways (Figure 5E).

### 3.6. The Lack of Dystrophin Results in Significant Changes in Several Proteins, including the Water Channel Protein, Aquaporin 4 (AQP4)

**Figure 6:**
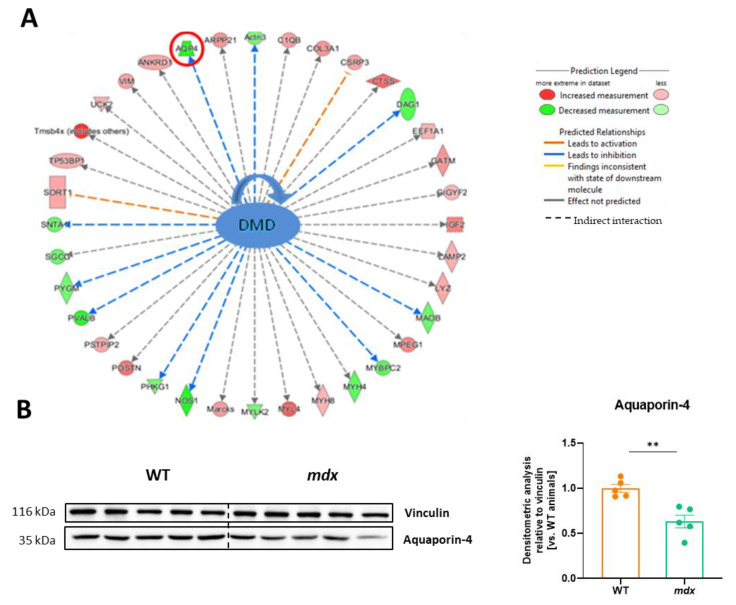
Aquaporin 4 (AQP4) abundance is decreased in dystrophin-deficient diaphragms. (**A**) Ingenuity pathway analysis (IPA) demonstrated changes in various dystrophin-associated proteins, including a decrease in the level of AQP4, which was confirmed by (**B**) Western blot (Figure S2) and its densitometric analysis, *n* = 5/group; results are shown as mean ± SEM; ** *p* < 0.01.

The IPA analysis revealed numerous proteins whose expression was affected by dystrophin deletion (Figure 6A). Among others, as expected, the abundance of the dystrophin-associated proteins α1-syntrophin (SNTA1) and δ-sarcoglycan (SGCG) was decreased, while that of collagen type III (COL3A1) or insulin-like growth factor 2 (IGF2) was increased. Furthermore, data for one of the affected proteins, AQP4, were also confirmed by a representative Western blot. Semi-quantification based on densitometry analysis showed an approximately 40% decrease in AQP4 expression in the *mdx* diaphragm (Figure 6B).

### 3.7. Decreased Expression of H_2_S-Generating Enzymes Is a Hallmark of DMD

**Figure 7:**
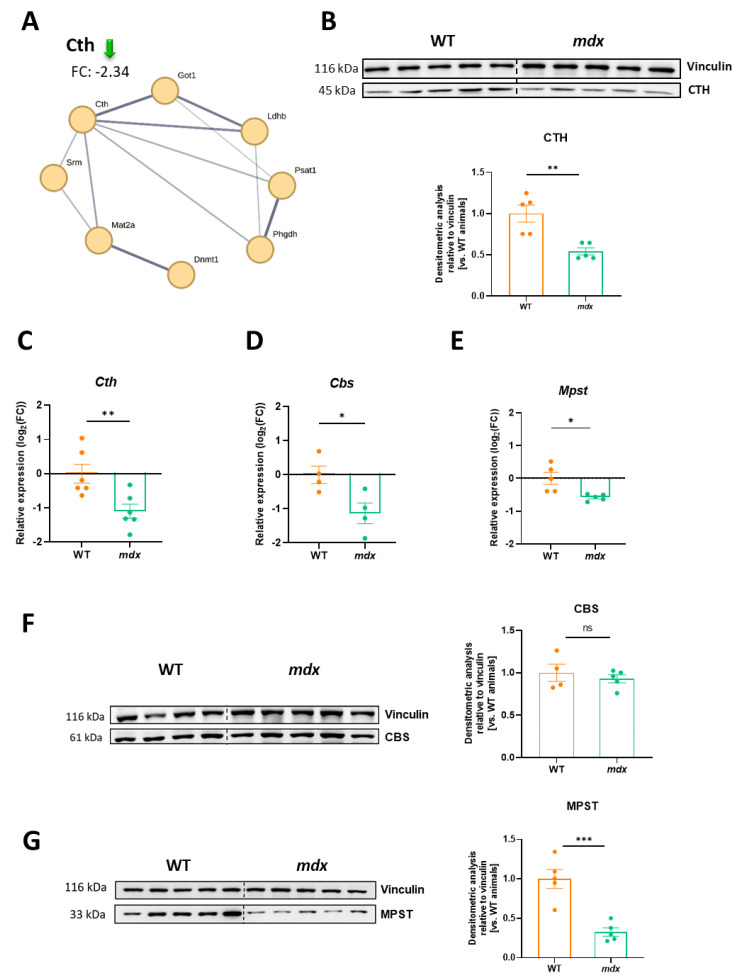
Cystathionine γ-lyase (CTH), cystathionine β-synthase (CBS), and 3-mercaptopyruvate sulfurtransferase (MPST) expression in dystrophin-deficient diaphragms. (**A**) A decrease in the CTH level found in the proteomic analysis was confirmed at the protein level as shown by (**B**) Western blotting (Figure S3) and its densitometric evaluation. A similar pattern was observed at the mRNA level not only for (**C**) *Cth* but also for other H_2_S-generating enzymes, (**D**) *Cbs* and (**E**) *Mpst.* (**F**) The protein level of CBS was not changed while (**G**) the potent decrease in MPST protein expression was demonstrated by Western blot (Figures S4 and S5) analysis and densitometric analysis; *n* = 4–5/group; results shown as mean ± SEM; * *p* < 0.05, ** *p* < 0.01, *** *p* < 0.001, ns–not significant.

Recently, we found decreased expression of H_2_S-generating enzymes in the muscles of 12-week-old dystrophin-deficient *mdx* mice [24]. In the present study, we also observed that among the highly altered proteins identified in the TMT analysis (Figure 2D), the abundance of cystathionine γ-lyase (CTH), responsible for H_2_S production from homocysteine, cystathionine, or L-cysteine was decreased. The level of CTH was approximately 2.3 times lower in the *mdx* diaphragm of young 6-week-old mice than in the diaphragm of the control counterparts (Figure 7A).

This result was confirmed by Western blot analysis, showing an almost 2-fold decrease as calculated based on densitometry (Figure 7B). Importantly, not only the CTH transcript (Figure 7C) but also the mRNA level of other H_2_S generating enzymes, *Cbs* (Figure 7D) and *Mpst* (Figure 7E), were decreased in *mdx* animals. However, CBS protein expression was not affected by the lack of dystrophin (Figure 7F), while Western blotting revealed a potently diminished expression of MPST protein in the dystrophic diaphragm (Figure 7G). In the proteomic analysis, MPST was also negatively regulated (fold-change threshold: −1.36) but did not meet our criteria for further consideration (fold-change threshold set at 1.5).

## 4. Discussion

Although the molecular mechanisms of Duchenne muscular dystrophy (DMD) are well described and the main cause, namely mutations in the *DMD* gene that encodes dystrophin, is known, the exact reason for why this leads to muscle breakdown and degeneration is not entirely clear. Dystrophin is not only a structural protein, being a molecular shock absorber, stretch sensor, and mechanical tension modulator [40], but it plays also nonmechanical, signaling roles, being a Ca^2+^ regulator and a modulator of nitric oxide (NO) production and ROS signaling [41,42]. Therefore, the multiple complex changes in cell membrane properties and cell mechanical defects, as well as the dysregulation of signaling pathways, might contribute to the muscle dysfunction caused by the absence of dystrophin.

Unfortunately, the disease is still incurable, and the search for new possible targets can help to discover therapeutic agents to ameliorate disease progression. Proteomic profiling serves as an ideal strategy for this purpose, as it is an excellent tool for analyzing differentially expressed proteins or, more precisely, proteoforms, between normal and diseased individuals. Given that the expression of the protein is dependent on RNA transcription, alternative splicing, and/or post-translational modifications (PTMs), various proteoforms might be formed from a given gene [43]. Therefore, the characterization of alterations in proteoforms and their dynamic PTMs is essential to a comprehensive study of muscle diseases like DMD. In our study, the use of label-free (LF) and tandem mass tag (TMT) methods allowed us to gather a larger pool of results and to confirm some of the obtained changes (proteins/proteoforms detected by both methods). However, the LF method served as a preliminary screening, and, in further analysis, we decided to focus on the TMT results due to their volume and complexity. In contrast to the label-free approach, TMT labeling enables the fractionation of peptides before LC-MS analysis while maintaining low variance between samples, thus increasing the number of identified proteins. On the other hand, isobaric labeling often suffers from ion interference and ratio distortion [44], which could have led to dystrophin detection in *mdx* mice. Another explanation for the detection of dystrophin in dystrophic animals may be related to the detection of the shorter isoform Dp71 or/and the presence of revertant fibers with restored dystrophin expression [45,46].

Over the last decade, many relevant studies have focused on detailed proteomic analyses of the skeletal muscles of dystrophic animals. Already in 2013, Rayavarapu et al. [47], by applying in vivo SILAC proteomics, had identified alterations in actin cytoskeleton signaling, the integrin-linked kinase pathway, mitochondrial energy metabolism, and calcium homeostasis in dystrophin-deficient *mdx* gastrocnemius. Matsumura et al., performed comparative research on the spared extraocular muscles, which do not show muscle degeneration, and the highly affected diaphragm using a label-based shotgun proteomic approach [48]. These studies indicated that the choice of the dystrophic muscle has a prominent impact on the proteomic results. Of note, the high heterogeneity of the muscle proteome also makes LC- or gel-based top-down approaches a promising direction for the analysis of dystrophy progression. Unlike bottom-up proteomics, these methods provide a protein-centric view, allowing for the analysis of changes in protein isoforms and PTM variants. The top-down approach gives a different perspective on alterations in and the diversity of proteins, which was proved to be valuable also for the study of muscular dystrophy [49,50,51]. Recent studies have also concentrated on combining the proteomic analysis with transcriptomic and metabolomic analyses [52], whole-transcriptome RNA sequencing, and mitochondrial proteomics [53], or on performing redox proteomics [54].

During degeneration, fibrosis and a significant functional impairment are known to occur in the *mdx* diaphragm in a way similar to that seen in human DMD limb muscles [19]. We and others [48,51,55,56,57] have chosen this muscle for proteomic analysis to examine in depth those differentially expressed proteins that regulate inflammation, fibrosis, contractile functions, components of the cytoskeletal network, and the extracellular matrix, as well as the immune response. We also intended to find new possible markers of DMD.

Gargan et al. [57] compared the young (3-month-old) versus the aged (15-month-old) diaphragm muscle of the *mdx-4cv* mouse model, in which a nonsense point mutation in exon 53 leads to premature translation termination and a lack of dystrophin. The drastic and age-related upregulation of proteins characteristic of tissue regeneration and fibrosis and markers of membrane repair, such as annexins and caveolins, has been evident in dystrophic animals. In a proteomic analysis of 10-month-old *mdx-4cv* mice, 289 decreased and 468 increased proteins were identified in the dystrophin-deficient diaphragm [51]. Among the differentially expressed proteins, dystrophin-associated proteins, such as α1-syntrophin, α-sarcoglycan, β-sarcoglycan, δ-sarcoglycan, and dystroglycan, were found. A similar lower abundance of proteins belonging to the dystrophin complex in the diaphragm of *mdx-4cv* mice was demonstrated by Gargan et al. [57]. In our hands, a reduction in sarcospan, α1-syntrophin, dystroglycan, α-sarcoglycan, β-sarcoglycan, and δ-sarcoglycan levels was also observed, indicating the mutant status of the *mdx* strain mice. We have also shown the consequences of dystrophin deficiency, as the enrichment of several important pathways, including the innate and adaptive immune system, ECM organization, protein translation, post-translational modification, programmed cell death, and cellular response to stress, was evident. Some of the detected proteins have already been mentioned in the literature, giving our results additional confirmatory status [58]. One of the examples could be the differences in the expression of various collagen isoforms. A detailed study of their abundance (and other ECM proteins) in dystrophic muscles and the muscle-associated layers of connective tissue was summarized recently [22]. Based on the increase in collagen V and collagen VI in the endomysium, collagen IV, matrisomal proteins at the level of the basal lamina, myotendinous-junction-enriched collagen XII, and tendon-enriched collagen I, it was suggested that these be used as biomarkers of dystrophinopathy-associated myofibrosis.

One of the highly abundant proteins detected in the LF approach in the dystrophic diaphragm was periostin (POSTN), a non-structural matricellular protein that accelerates inflammation or fibrosis. Our finding fits well with previous reports demonstrating the drastic increase in POSTN levels in muscular dystrophy. Initially, the correlation between the significantly upregulated POSTN level (identified by the label-free MS analysis) in the dystrophin-deficient *mdx-4cv* diaphragm and progressive fibrosis of dystrophic tissue was revealed by Holland et al. [59], and it was then also shown in the dystrophic heart [60]. As periostin expression increases potently during muscle regeneration [61], it may represent a useful indicator of myofibrotic changes in DMD (reviewed in [22]).

We have to also stress that in DMD, the level of other cytoskeletal proteins could be highly changed, including that of tubulin, vimentin, and vinculin [59]. We also identified tubulin as a protein with higher abundance in the *mdx* diaphragm; however, the vinculin level was much more stable, as indicated also by our Western blot data. Nevertheless, one has to remember that this particular protein might not always be an adequate loading control when studying dystrophic pathology, and its usefulness has to be checked and confirmed empirically.

In addition to dystrophin-associated and ECM proteoforms, another highly reduced protein in the dystrophic diaphragm was aquaporin-4 (AQP4), the main water channel of the neuromuscular system. Previous studies showed markedly reduced expression of the AQP4 protein in the diaphragms of 3-month-old dystrophic mice [62], as well as in the quadriceps femoris muscle [63]. Frigeri et al. [64] performed a more detailed analysis, demonstrating the reduction in AQP4 at the sarcolemmal level of skeletal-muscle fast fibers, especially type IIB fibers, and in the brain but not in the stomach and kidney. Importantly, an age-dependent effect was observed, and only a 20% reduction was found in young 2-month-old mice, while in 1-year-old animals, the differences reached 90% [64]. AQP4 was also downregulated in the tibialis anterior muscles from two murine DMD models (*mdx* and *mdx52*, with deletion of *Dmd* exon 52, which leads to the absence of the Dp260 and Dp140 isoforms in addition to Dp427) at three ages (8, 16, and 80 weeks of age) [65]. Thus, the differential expression of AQP4 between non-diseased and dystrophic individuals seems to be a universal phenomenon. Importantly, in human patients with DMD, a reduction in AQP4 level, as assessed by immunofluorescence, was also evident [66]. However, in the milder form of muscular dystrophy, Becker muscular dystrophy (BMD), differences were found only in 50% of the analyzed muscular biopsies [66].

To the best of our knowledge, we for the first time, using proteomic analysis, have shown a decrease in the expression of the enzyme responsible for hydrogen sulfide (H_2_S) generation. The TMT-based method revealed the differential expression of cystathionine γ-lyase (CTH) between WT and *mdx* mice. We confirmed this result using real-time PCR and Western blot methods, and we also extended our study to the evaluation of other enzymes responsible for H_2_S production. In addition to the CTH level, we demonstrated a decrease in the expression of another H_2_S-generating enzyme, MPST, in the dystrophic diaphragm. Little is still known about H_2_S signaling in DMD. This cytoprotective molecule, through its antioxidant, anti-inflammatory, antifibrotic, and anti-apoptotic activities, as well as cardioprotective effects, represents a justified target in the treatment of DMD [23]. Decreased abundance of the expression of H_2_S-generating enzymes in the dystrophic diaphragm, as shown in the present work, and in other muscles, as indicated in recent reports [24,25,27], may have strong implications for potential therapies to combat muscular disorders. Nevertheless, to draw any conclusion, additional cell-specific experiments are required due to the significant differences in the cell composition between healthy and dystrophic diaphragms. Moreover, it would be of great importance to identify the main cellular source of these enzymes in the diseased tissue. Consequently, current studies are focusing on counteracting the H_2_S deficiency in dystrophic animals by applying H_2_S donors. Such therapies improved the progression of DMD in *mdx* mice [24,25,26] and in the *C. elegans* model of dystrophy [27]. We have also recently found a beneficial effect of the fast-releasing H_2_S donor, sodium hydrosulfide (NaHS), on inflammatory, angiogenic, and autophagy-related factors in the mouse model of DMD [24]. Together, these results indicate that enzymes in the transsulfuration pathway contribute to maintaining the proper functioning of the muscular system.

## 5. Conclusions

In conclusion, the lack of dystrophin leads to changes in numerous proteins in the diaphragm. Importantly, the alterations in the level of H_2_S-generating enzymes already in 6-week-old animals suggest that this gaseous mediator is an important regulator of DMD progression.

## 6. Study Limitations and Future Perspectives

First, since many proteomic analyses of the dystrophic phenotype, including those focusing on a wide range of ages from young to very old *mdx* mice and those using related murine models investigating both musculature in general and, more specifically, the dystrophic diaphragm muscle have been published, we were not able to discuss and cite all publications. Second, we used the *mdx* mouse, which differs in disease severity compared with other murine DMD models and human pathology. Nevertheless, as we found differences in the expression of H_2_S-generating enzymes in such a relatively mild model of DMD pathology, it might be expected that in more affected mice, similar or even exacerbated alterations in the levels of this cytoprotective gaseous molecule may be detected. We are aware that this finding needs to be elaborated further in future in vivo studies. Finally, it would be important to validate the observations in human dystrophic muscles.

## Figures and Tables

**Table 1:** Sequences of primers used for the determination of gene expression by qRT-PCR.

Gene	Sequence 5′-3′
*Casp3*	F: TGTCATCTCGCTCTGGTACG
	R: AAATGACCCCTTCATCACCA
*Cbs*	F: CCTAATTCTCACATTCTGGAC
	R: GACACCGATGATTTTACAGC
*Col1a1*	F: CGATCCAGTACTCTCCGCTCTTCC
	R: ACTACCGGGCCGATGATGCTAACG
*Col3a1*	F: ATCTATGAATGGTGGTTTTCA
	R: TTTTGCAGTGGTATGTAATGT
*Cth*	F: GAAAAGGTTGTTTATCCTGGG
	R: CTTGATGTAGAAACTGACCATC
*Eef2*	F: AGAACATATTATTGCTGGCG
	R: AACAGGGTCAGATTTCTTG
*Mpst*	F: CGTCCTACTTGCTTTTCTC
	R: CAGAGCTCGGAAAAGTTG
*Spp1*	F: CCATCTCAGAAGCAGAATCTCCTT
	R: GGTCATGGCTTTCATTGGAATT

## Data Availability

The authors confirm the availability of all data generated or analyzed in this manuscript. The mass spectrometry proteomics data have been deposited at the ProteomeXchange Consortium via the PRIDE [67] partner repository with the dataset identifier PXD040527.

## Supplementary Materials

The following supporting information can be downloaded at: https://www.mdpi.com/article/10.3390/biom13111648/s1, Table S1: Protein identification in *mdx* vs. WT mice using the LF approach. Table S2: Protein identification in *mdx* vs. WT mice using the TMT approach. Table S3: List of the enriched pathways in the *mdx* vs. WT mouse analysis. Figure S1: Original images of Figure 2E; Figure S2: Original images of Figure 6B; Figure S3: Original images of Figure 7B; Figure S4: Original images of Figure 7F; Figure S5: Original images of Figure 7G.
