# Supplementary material for: Proteome Profiling of the Dystrophic mdx Mice Diaphragm
Source: Biomolecules. 2023 Nov 13;13(11):1648. doi: 10.3390/biom13111648 (PMC10669179; doi:10.3390/biom13111648)
Supplement: Supplementary file 1 [file biomolecules-13-01648-s001.zip › Table S3.pdf]

Table S1. List of the enriched pathways in the *mdx* vs. WT analysis. Created based on the proteomics data (protein ID changed for gene ID for the purpose of analysis) using the Search Tool for the Retrieval of Interacting Genes/Proteins (STRING) database (and the incorporated Reactome and KEGG databases). FDR < 0.05

#term ID	term description	changed genes	all genes in the pathway	strength	FDR	Changed genes
**MMU-168256**	Immune System	154	1621	0.35	7.06e-18	Itgb2,Tubb6,Col1a1,Tubb5,Plaur,Calr,Cstb,Cd44,Vav1,Ctsb,Arpc2,Srp14,Galns,Cybb,Ctss,Ncf1,Ctsz,Myh9,Serpinb6a,Vtn,Cyba,Timp2,Atp6v1e1,Psme3,Apaf1,Hsp90b1,Tbk1,Lgmn,Npc2,Ddx41,Lpcat1,Hexb,Plau,Ripk3,Atp6v1c1,Igf2r,Tapbp,Csf1r,P4hb,Pa2g4,Gusb,Casp1,Casp8,Anapc2,Pdia3,Fabp5,Ptpn1,Ptx3,S100a11,Vcam1,Tlr2,Bcl10,Fgr,Anapc4,Slc15a4,Ctsc,Copb1,Pak1,Asah1,Man2b1,Cotl1,Ifi30,Csk,Ltf,Nckap1l,Rap2b,C1qb,Eef1a1,Grn,Dok3,Vat1,C1qa,Cul7,Itpr2,Cnpy3,Ckap4,Epx,Hk3,Lamp2,Stbd1,Glb1,Pstpip1,Tubb2a,Plekho2,Iqgap2,Itgam,Itgb5,H2Eb1,Hp,Tubb2b,Prcp,Glmn,Impdh1,Tax1bp1,Plcg2,Wasf2,Klc1,Arpc1b,Card11,Col3a1,Fyb,Dock2,Casp3,Cdc27,Sec31a,Ncf4,Cd74,Ceacam1,Hmha1,Mrc2,Sirpa,Arpc3,Nos1,Cd63,Stat2,Psap,Cap1,Camk2d,Clta,Trim25,Gmfg,Ap1s1,Camp,Mlec,Eif4e2,Pja1,Atp6v1a,Tmem173,S100a9,Pld3,Syk,Lgals3,Arpc4,Dsp,Myo5a,Fbxo30,Ppia,Abi1,Ctsd,Cdc23,Stxbp2,Panx1,Iqgap1,Ptpn6,Myo9b,Ripk1,C5ar1,Keap1,Psmb9,Pja2,Actr3,Ptprc,Bin2,Ncf2
**MMU-392499**	Metabolism of proteins	147	1609	0.33	1.33e-15	Galnt1,Rpl13,Tubb6,Pabpc1,Plaur,Rps11,Calr,Rpl8,Dnmt1,Ctr9,Rcn1,Rps9,Rplp1,Rps18,Srp14,Ctsz,Tnip1,Cog1,Psme3,Hdac2,Rab32,Hsp90b1,Gfpt2,Matn3,Lman2,Arf4,Rpl24,P4hb,Rps26,Bet1l,Hgs,Pdia3,Pcna,Chgb,Bcl10,Alpl,Rpl6,Rpl28,Ctsc,Copb1,Rnf40,Nucb1,Rps4x,Ces1d,Arcn1,Rpl4,Copb2,Sec23ip,Npl,Spon1,Rpl21,Rab8b,Eef1a1,Spon2,Rpl22l1,Rps27l,Rpl37,Usp28,Cul7,Ckap4,Pdia6,Rps23,Fn1,Tmed2,Glb1,Tmed3,Rpl37a,Tubb2a,Rab33b,Arfgap3,Rab13,Rpl7,Commd7,Hist1h2af,Rpl26,Rps12,Rps7,Npm1,Tubb2b,Rfwd2,Rpl17,Rpl15,Rps25,Rpl36,Rpl35,Rpl29,Rpl3,Rpl32,Rpl5,Rplp2,Man2a1,Rpl34,Lgals1,Cd109,Sec31a,Ccdc8,Golm1,Rpl23a,Rpl11,Hdac1,Rps8,Stam2,Rps6,Rpl7a,Stam,Igf2,Tnc,Dpm3,Rps16,Rps19,Top1,Vcan,Trp53bp1,Hnrnpc,Spp1,Obsl1,Copg1,Gfpt1,Rpl9,Rps28,Fstl1,Rpl35a,Prkcsh,Renbp,Rpl13a,Eef1b2,Fbxo30,Rps15a,Rps20,Dda1,Ctsd,Mcfd2,Rpl27a,Galnt4,Rps24,Mdga1,Ripk1,Dag1,Keap1,Nup50,Rpl14,Wac,Apoe,Psmb9,Spcs3,Rpl31,Nop58

**MMU-8953854**	Metabolism of RNA	121	556	0.71	1.84e-41	Rpl13,Pabpc1,Sf3a1,Rps11,Rpl8,Rps9,Wtap,Khsrp,Rplp1,Prpf31,Rps18,Prpf3,Rrp7a,Pelp1,Psme3,Ddx39,Snrpf,Ftsj3,Mnat1,Smn1,Ppwd1,Pabpn1,Phf5a,Rpl24,Rbm22,Rps26,Wdr75,Ncl,Cstf3,Ctnnbl1,Exosc9,Ncbp1,Ebna1bp2,Sf3a3,Srsf9,Rpl6,Ddx47,Rpl28,Mphosph10,Cstf2,Rps4x,Mphosph6,Rpl4,Tsr1,Cnot3,Wdr18,Rpl21,Ddx21,Rpl22l1,Thoc2,Rps27l,Rpl37,Cpsf2,Utp15,Wdr43,Rpp38,Rps23,Rpl37a,Usp39,Utp18,Rpl7,Pdcd11,Rpl26,Rps12,Rps7,Prpf38a,Rpl17,Rpl15,Rps25,Rpl36,Rpl35,Rpl29,Rpl3,Rpl32,Rpl5,Rplp2,Gtf2f2,Rpl34,Isy1,Utp3,Nhp2l1,Puf60,Rpl23a,Rpl11,Rps8,Rps6,Set,Rpl7a,Sf3b5,Cnot2,Fus,Rngtt,Rps16,Rps19,Cnot8,Polr2c,Papola,Pes1,Srsf5,Hnrnpc,Rpl9,Rps28,Rpl35a,Pqbp1,Rpl13a,Snrpa1,Srsf6,Rps15a,Rps20,Sf1,Rpl27a,Srrm1,Bud31,Rps24,Hnrnpf,Nup50,Rpl14,Tex10,Psmb9,Rpl31,Nop58
**MMU-168249**	Innate Immune System	114	949	0.45	1.14e-19	Itgb2,Tubb5,Plaur,Cstb,Cd44,Vav1,Ctsb,Arpc2,Srp14,Galns,Cybb,Ctss,Ncf1,Ctsz,Myh9,Serpinb6a,Vtn,Cyba,Timp2,Atp6v1e1,Psme3,Apaf1,Hsp90b1,Tbk1,Lgmn,Npc2,Ddx41,Lpcat1,Hexb,Plau,Ripk3,Atp6v1c1,Igf2r,Pa2g4,Gusb,Casp1,Casp8,Fabp5,Ptx3,S100a11,Tlr2,Bcl10,Fgr,Slc15a4,Ctsc,Copb1,Pak1,Asah1,Man2b1,Cotl1,Ltf,Nckap1l,Rap2b,C1qb,Eef1a1,Grn,Dok3,Vat1,C1qa,Cnpy3,Ckap4,Epx,Hk3,Lamp2,Stbd1,Glb1,Pstpip1,Plekho2,Iqgap2,Itgam,Hp,Prcp,Impdh1,Tax1bp1,Plcg2,Wasf2,Arpc1b,Card11,Dock2,Ncf4,Ceacam1,Hmha1,Sirpa,Arpc3,Nos1,Cd63,Psap,Cap1,Gmfg,Camp,Mlec,Atp6v1a,Tmem173,S100a9,Pld3,Syk,Lgals3,Arpc4,Dsp,Myo5a,Ppia,Abi1,Ctsd,Panx1,Iqgap1,Ptpn6,Myo9b,Ripk1,C5ar1,Psmb9,Actr3,Ptprc,Bin2,Ncf2
**MMU-597592**	Post-translational protein modification	90	1254	0.23	8.52e-05	Galnt1,Tubb6,Plaur,Calr,Dnmt1,Ctr9,Rcn1,Ctsz,Tnip1,Cog1,Psme3,Hdac2,Rab32,Hsp90b1,Gfpt2,Matn3,Lman2,Arf4,P4hb,Bet1l,Hgs,Pdia3,Pcna,Chgb,Bcl10,Alpl,Ctsc,Copb1,Rnf40,Nucb1,Arcn1,Copb2,Sec23ip,Npl,Spon1,Rab8b,Eef1a1,Spon2,Usp28,Cul7,Ckap4,Pdia6,Fn1,Tmed2,Glb1,Tmed3,Tubb2a,Rab33b,Arfgap3,Rab13,Commd7,Hist1h2af,Npm1,Tubb2b,Rfwd2,Man2a1,Lgals1,Cd109,Sec31a,Ccdc8,Golm1,Hdac1,Stam2,Stam,Tnc,Dpm3,Top1,Vcan,Trp53bp1,Hnrnpc,Spp1,Obsl1,Copg1,Gfpt1,Fstl1,Prkcsh,Renbp,Fbxo30,Dda1,Mcfd2,Galnt4,Mdga1,Ripk1,Dag1,Keap1,Nup50,Wac,Apoe,Psmb9,Nop58
**MMU-6791226**	Major pathway of rRNA processing in the nucleolus and cytosol	73	171	1.0	4.85e-40	Rpl13,Rps11,Rpl8,Rps9,Rplp1,Rps18,Rrp7a,Pelp1,Ftsj3,Rpl24,Rps26,Wdr75,Ncl,Exosc9,Ebna1bp2,Rpl6,Ddx47,Rpl28,Mphosph10,Rps4x,Mphosph6,Rpl4,Tsr1,Wdr18,Rpl21,Ddx21,Rpl22l1,Rps27l,Rpl37,Utp15,Wdr43,Rpp38,Rps23,Rpl37a,Utp18,Rpl7,Pdcd11,Rpl26,Rps12,Rps7,Rpl17,Rpl15,Rps25,Rpl36,Rpl35,Rpl29,Rpl3,Rpl32,Rpl5,Rplp2,Rpl34,Utp3,Nhp2l1,Rpl23a,Rpl11,Rps8,Rps6,Rpl7a,Rps16,Rps19,Pes1,Rpl9,Rps28,Rpl35a,Rpl13a,Rps15a,Rps20,Rpl27a,Rps24,Rpl14,Tex10,Rpl31,Nop58
**MMU-679**	Neutrophil degranulation	73	519	0.52	1.90e-	Itgb2,Tubb5,Plaur,Cstb,Cd44,Ctsb,Srp14,Galns,Cybb,Ctss,Ctsz,Serpinb6a,Cyba,Timp2,Apaf1,Npc2,Lpcat1,Hexb,Plau,Igf2r,Pa2g4,Gusb,Fabp5,Ptx3,S100a11,Tlr2,Fgr,Slc15a4,Ctsc,Copb1,Asah1,Man2b1,Cotl1,Ltf,Nckap1l,Rap2b,Eef1a1,Grn,Dok3,Vat1,Ckap4,Epx,Hk3,Lamp2,Stbd1,Glb1,Plekho2,Iqgap2,Itgam,Hp,Prcp,Impdh1,Dock2,Ceacam1,Hmha1,Sirpa,Cd63,Psap,C

**8695**					15	ap1,Gmfg,Camp,Mlec,Tmem173,S100a9,Lgals3,Dsp,Ppia,Ctsd,Iqgap1,Ptpn6,C5ar1,Ptprc,Bin2
**MMU-109582**	Hemostasis	59	524	0.43	7.95e-09	Itgb2,Tubb6,Col1a1,Plaur,Cd44,Vav1,Itih3,Apbb1ip,Serpinb6a,Sparc,Hdac2,Habp4,Plau,Itga5,Pcyox1l,Dock8,Grb14,Ptpn1,Pdpn,Fgr,P2rx4,Csk,Atp1b3,Islr,Itpr2,Lamp2,Fn1,Tubb2a,Lhfpl2,Itgam,Pla2g4a,Tubb2b,Prcp,Dock10,Plcg2,Klc1,Dock2,Cd109,Cd74,Ceacam1,Sirpa,Nos1,Hdac1,Akap10,Plek,Cd63,Psap,Igf2,Tagln2,Kdm1a,Cbx5,Syk,Ppia,Cd84,Stxbp2,Ptpn6,Slc3a2,Glg1,Jmjd1c
**MMU-72766**	Translation	54	223	0.76	4.46e-20	Rpl13,Pabpc1,Rps11,Rpl8,Rps9,Rplp1,Rps18,Srp14,Rpl24,Rps26,Rpl6,Rpl28,Rps4x,Rpl4,Rpl21,Eef1a1,Rpl22l1,Rps27l,Rpl37,Rps23,Rpl37a,Rpl7,Rpl26,Rps12,Rps7,Rpl17,Rpl15,Rps25,Rpl36,Rpl35,Rpl29,Rpl3,Rpl32,Rpl5,Rplp2,Rpl34,Rpl23a,Rpl11,Rps8,Rps6,Rpl7a,Rps16,Rps19,Rpl9,Rps28,Rpl35a,Rpl13a,Eef1b2,Rps15a,Rps20,Rpl27a,Rps24,Rpl14,Rpl31
**MMU-5653656**	Vesicle-mediated transport	54	594	0.33	2.44e-05	Galnt1,Tubb6,Sh3gl1,Calr,Pafah1b3,Arpc2,Ctsz,Sparc,Cog1,Rab32,Hsp90b1,Lman2,Arf4,Igf2r,Ap3s1,Msr1,Bet1l,Hgs,Acbd3,Ctsc,Copb1,Arcn1,Copb2,Sec23ip,Rab8b,Stab1,Tmed2,Tmed3,Tubb2a,Rab33b,Arfgap3,Rab3gap2,Rab13,Pla2g4a,Hp,Tubb2b,Klc1,Gcc1,Tpd52,Sec31a,Cttn,Arpc3,Sort1,Stam2,Stam,Cyth2,Clta,Ap1s1,Fnbp1,Copg1,Arpc4,Mcfd2,Apoe,Actr3
**MMU-975956**	Nonsense Mediated Decay (NMD) independent of the Exon Junction Complex (EJC)	52	90	1.14	2.37e-33	Rpl13,Pabpc1,Rps11,Rpl8,Rps9,Rplp1,Rps18,Rpl24,Rps26,Ncbp1,Rpl6,Rpl28,Rps4x,Rpl4,Rpl21,Rpl22l1,Rps27l,Rpl37,Rps23,Rpl37a,Rpl7,Rpl26,Rps12,Rps7,Rpl17,Rpl15,Rps25,Rpl36,Rpl35,Rpl29,Rpl3,Rpl32,Rpl5,Rplp2,Rpl34,Rpl23a,Rpl11,Rps8,Rps6,Rpl7a,Rps16,Rps19,Rpl9,Rps28,Rpl35a,Rpl13a,Rps15a,Rps20,Rpl27a,Rps24,Rpl14,Rpl31
**MMU-975957**	Nonsense Mediated Decay (NMD) enhanced by the Exon Junction Complex (EJC)	52	110	1.05	2.93e-30	Rpl13,Pabpc1,Rps11,Rpl8,Rps9,Rplp1,Rps18,Rpl24,Rps26,Ncbp1,Rpl6,Rpl28,Rps4x,Rpl4,Rpl21,Rpl22l1,Rps27l,Rpl37,Rps23,Rpl37a,Rpl7,Rpl26,Rps12,Rps7,Rpl17,Rpl15,Rps25,Rpl36,Rpl35,Rpl29,Rpl3,Rpl32,Rpl5,Rplp2,Rpl34,Rpl23a,Rpl11,Rps8,Rps6,Rpl7a,Rps16,Rps19,Rpl9,Rps28,Rpl35a,Rpl13a,Rps15a,Rps20,Rpl27a,Rps24,Rpl14,Rpl31

**MMU-1280218**	Adaptive Immune System	52	701	0.24	0.00040	Itgb2,Tubb6,Col1a1,Calr,Vav1,Ctsb,Cybb,Ctss,Ncf1,Cyba,Psme3,Lgmn,Tapbp,Anapc2,Pdia3,Vcam1,Bcl10,Anapc4,Ctsc,Pak1,Ifi30,Csk,Cul7,Itpr2,Tubb2a,Itgb5,H2-Eb1,Tubb2b,Glmn,Plcg2,Klc1,Card11,Col3a1,Fyb,Cdc27,Sec31a,Ncf4,Cd74,Mrc2,Clta,Ap1s1,Pja1,Syk,Fbxo30,Ctsd,Cdc23,Ptpn6,Keap1,Psmb9,Pja2,Ptprc,Ncf2
**MMU-1799339**	SRP-dependent cotranslational protein targeting to membrane	51	88	1.14	8.24e-33	Rpl13,Rps11,Rpl8,Rps9,Rplp1,Rps18,Srp14,Rpl24,Rps26,Rpl6,Rpl28,Rps4x,Rpl4,Rpl21,Rpl22l1,Rps27l,Rpl37,Rps23,Rpl37a,Rpl7,Rpl26,Rps12,Rps7,Rpl17,Rpl15,Rps25,Rpl36,Rpl35,Rpl29,Rpl3,Rpl32,Rpl5,Rplp2,Rpl34,Rpl23a,Rpl11,Rps8,Rps6,Rpl7a,Rps16,Rps19,Rpl9,Rps28,Rpl35a,Rpl13a,Rps15a,Rps20,Rpl27a,Rps24,Rpl14,Rpl31
**MMU-156827**	L13a-mediated translational silencing of Ceruloplasmin expression	51	107	1.05	6.93e-30	Rpl13,Pabpc1,Rps11,Rpl8,Rps9,Rplp1,Rps18,Rpl24,Rps26,Rpl6,Rpl28,Rps4x,Rpl4,Rpl21,Rpl22l1,Rps27l,Rpl37,Rps23,Rpl37a,Rpl7,Rpl26,Rps12,Rps7,Rpl17,Rpl15,Rps25,Rpl36,Rpl35,Rpl29,Rpl3,Rpl32,Rpl5,Rplp2,Rpl34,Rpl23a,Rpl11,Rps8,Rps6,Rpl7a,Rps16,Rps19,Rpl9,Rps28,Rpl35a,Rpl13a,Rps15a,Rps20,Rpl27a,Rps24,Rpl14,Rpl31
**MMU-72737**	Cap-dependent Translation Initiation	51	115	1.02	8.64e-29	Rpl13,Pabpc1,Rps11,Rpl8,Rps9,Rplp1,Rps18,Rpl24,Rps26,Rpl6,Rpl28,Rps4x,Rpl4,Rpl21,Rpl22l1,Rps27l,Rpl37,Rps23,Rpl37a,Rpl7,Rpl26,Rps12,Rps7,Rpl17,Rpl15,Rps25,Rpl36,Rpl35,Rpl29,Rpl3,Rpl32,Rpl5,Rplp2,Rpl34,Rpl23a,Rpl11,Rps8,Rps6,Rpl7a,Rps16,Rps19,Rpl9,Rps28,Rpl35a,Rpl13a,Rps15a,Rps20,Rpl27a,Rps24,Rpl14,Rpl31
**MMU-72689**	Formation of a pool of free 40S subunits	50	97	1.09	2.29e-30	Rpl13,Rps11,Rpl8,Rps9,Rplp1,Rps18,Rpl24,Rps26,Rpl6,Rpl28,Rps4x,Rpl4,Rpl21,Rpl22l1,Rps27l,Rpl37,Rps23,Rpl37a,Rpl7,Rpl26,Rps12,Rps7,Rpl17,Rpl15,Rps25,Rpl36,Rpl35,Rpl29,Rpl3,Rpl32,Rpl5,Rplp2,Rpl34,Rpl23a,Rpl11,Rps8,Rps6,Rpl7a,Rps16,Rps19,Rpl9,Rps28,Rpl35a,Rpl13a,Rps15a,Rps20,Rpl27a,Rps24,Rpl14,Rpl31
**MMU-72706**	GTP hydrolysis and joining of the 60S ribosomal subunit	50	108	1.04	7.42e-	Rpl13,Rps11,Rpl8,Rps9,Rplp1,Rps18,Rpl24,Rps26,Rpl6,Rpl28,Rps4x,Rpl4,Rpl21,Rpl22l1,Rps27l,Rpl37,Rps23,Rpl37a,Rpl7,Rpl26,Rps12,Rps7,Rpl17,Rpl15,Rps25,Rpl36,Rpl35,Rpl29,Rpl3,Rpl32,Rpl5,Rplp2,Rpl34,Rpl23a,Rpl11,Rps8,Rps6,Rpl7a,Rps16,Rps19,Rpl9,Rps28,Rpl35a,Rpl13a,Rps15a,Rps20,Rpl27a,Rps24,Rpl14,Rpl31

					29	
MMU-199991	Membrane Trafficking	47	560	0.3	0.00071	Galnt1,Tubb6,Sh3gl1,Pafah1b3,Arpc2,Ctsz,Cog1,Rab32,Lman2,Arf4,Igf2r,Ap3s1,Bet1l,Hgs,Acbd3,Ctsc,Copb1,Arcn1,Copb2,Sec23ip,Rab8b,Tmed2,Tmed3,Tubb2a,Rab33b,Arfgap3,Rab3gap2,Rab13,Pla2g4a,Tubb2b,Klc1,Gcc1,Tpd52,Sec31a,Cttn,Arpc3,Sort1,Stam2,Stam,Cyth2,Clta,Ap1s1,Fnbp1,Copg1,Arpc4,Mcfd2,Actr3
MMU-1474244	Extracellular matrix organization	45	295	0.56	2.53e-10	Itgb2,Col1a1,Cd44,Ctsb,Htra1,P4ha1,Ctss,Vtn,Timp2,Sparc,P4ha2,Plod1,Matn3,Itga5,Leprel2,P4hb,Col5a1,Vcam1,Emilin1,Pcolce,Acan,Mmp2,Ppib,Colgalt1,Fbln2,Fn1,Itgam,Itgb5,Sh3pxd2a,Crtap,Col5a2,Capn6,Col3a1,Ceacam1,Matn1,Lepre1,Tnc,Vcan,Col14a1,Spp1,Ctsd,Plod2,Serpinh1,Lox,Dag1
MMU-194315	Signaling by Rho GTPases	43	381	0.43	2.05e-06	Tubb6,Rhoc,Vav1,Arpc2,Cybb,Ncf1,Myh9,Cyba,Ywhah,Evl,Hgs,Arhgef2,Arhgdib,Pak1,Nckap1l,Gmip,Fmnl1,Arhgap15,Rhoj,Tubb2a,Iqgap2,Rcc2,Tubb2b,Wasf2,Klc1,Arpc1b,Arhgap24,Ncf4,Hmha1,Mad2l1,Arpc3,Myh10,Gopc,Arap1,Fgd3,Arhgap25,S100a9,Arpc4,Abi1,Iqgap1,Myo9b,Actr3,Ncf2
MMU-9006934	Signaling by Receptor Tyrosine Kinases	39	418	0.34	0.000045	Col1a1,Sh3gl1,Vav1,Cybb,Ncf1,Cyba,Atp6v1e1,Thbs4,Atp6v1c1,Hgs,Col5a1,Ptpn1,Ncbp1,Ptpn12,Pak1,Csk,Nckap1l,Cilp,Fn1,Chd4,Wasf2,Col5a2,Col3a1,Gtf2f2,Tia1,Ncf4,Stam2,Stam,Igf2,Clta,Polr2c,Spp1,Nrp2,Atp6v1a,Abi1,Ptpn6,Hnrnpf,Ncf2,Erbb2ip
MMU-72203	Processing of Capped Intron-Containing Pre-mRNA	38	234	0.58	2.15e-09	Sf3a1,Wtap,Prpf31,Prpf3,Ddx39,Snrpf,Ppwd1,Pabpn1,Phf5a,Rbm22,Cstf3,Ctnnbl1,Ncbp1,Sf3a3,Srsf9,Cstf2,Thoc2,Cpsf2,Usp39,Prpf38a,Gtf2f2,Isy1,Nhp2l1,Puf60,Sf3b5,Fus,Polr2c,Papola,Srsf5,Hnrnpc,Pqbp1,Snrpa1,Srsf6,Sf1,Srrm1,Bud31,Hnrnpf,Nup50
MMU-	Cellular responses to external stimuli	35	397	0.32	0.00	Tubb6,Ehmt2,Cybb,Ncf1,Cyba,Atp6v1e1,Psme3,Asf1a,Gpx8,Atp6v1c1,P4hb,Cdk2,Anapc2,Gpx7,Dnajc2,Anapc4,Mt2,Eef1a1,Ptges3,Tubb2a,Hist1h1a,Hist1h2af,Tubb2b,Hist1h1b,Cdc27,Ncf4,Rbbp4,Sirt1,Camk2d,Atp6v1a,Cdc23,Cdkn1c,Nup50,Psmb9,Ncf2

**8953897**					26	
**MMU-72163**	mRNA Splicing - Major Pathway	34	178	0.66	5.51e-10	Sf3a1,Prpf31,Prpf3,Snrpf,Ppwd1,Pabpn1,Phf5a,Rbm22,Cstf3,Ctnnbl1,Ncbp1,Sf3a3,Srsf9,Cstf2,Cpsf2,Usp39,Prpf38a,Gtf2f2,Isy1,Nhp2l1,Puf60,Sf3b5,Fus,Polr2c,Papola,Srsf5,Hnrnpc,Pqbp1,Snrpa1,Srsf6,Sf1,Srrm1,Bud31,Hnrnpf
**MMU-2262752**	Cellular responses to stress	34	395	0.31	0.00047	Tubb6,Ehmt2,Cybb,Ncf1,Cyba,Atp6v1e1,Psme3,Asf1a,Gpx8,Atp6v1c1,P4hb,Cdk2,Anapc2,Gpx7,Dnajc2,Anapc4,Eef1a1,Ptges3,Tubb2a,Hist1h1a,Hist1h2af,Tubb2b,Hist1h1b,Cdc27,Ncf4,Rbbp4,Sirt1,Camk2d,Atp6v1a,Cdc23,Cdkn1c,Nup50,Psmb9,Ncf2
**MMU-195258**	RHO GTPase Effectors	30	246	0.46	4.77e-05	Tubb6,Rhoc,Arpc2,Cybb,Ncf1,Myh9,Cyba,Ywhah,Evl,Pak1,Nckap1l,Fmnl1,Tubb2a,Iqgap2,Rcc2,Tubb2b,Wasf2,Klc1,Arpc1b,Ncf4,Mad2l1,Arpc3,Myh10,Gopc,S100a9,Arpc4,Abi1,Iqgap1,Actr3,Ncf2
**MMU-446203**	Asparagine N-linked glycosylation	28	263	0.4	0.00082	Tubb6,Calr,Ctsz,Cog1,Gfpt2,Lman2,Arf4,Bet1l,Pdia3,Ctsc,Copb1,Arcn1,Copb2,Sec23ip,Npl,Tmed2,Glb1,Tmed3,Tubb2a,Arfgap3,Tubb2b,Man2a1,Sec31a,Dpm3,Copg1,Gfpt1,Renbp,Mcfd2
**MMU-76002**	Platelet activation, signaling and aggregation	26	253	0.39	0.0023	Col1a1,Vav1,Itih3,Apbb1ip,Sparc,Habp4,Pcyox1l,Ptpn1,Pdpn,Csk,Islr,Itpr2,Lamp2,Fn1,Lhfpl2,Pla2g4a,Plcg2,Cd109,Plek,Cd63,Psap,Igf2,Tagln2,Syk,Stxbp2,Ptpn6
**MMU-**	Axon guidance	25	278	0.33	0.01	Tubb6,Rhoc,Arpc2,Evl,Itga5,Dpysl3,Col5a1,Vasp,Pak1,Mmp2,Tubb2a,Tubb2b,Enah,Arpc1b,Col5a2,Col3a1,Arpc3,Clta,Msn,Arpc4,Myo9b,Dag1,Ncam1,Actr3,Ptprc

422475					52	
MMU-6811442	Intra-Golgi and retrograde Golgi-to-ER traffic	22	186	0.45	0.0014	Galnt1,Tubb6,Pafah1b3,Cog1,Arf4,Igf2r,Bet1l,Copb1,Arcn1,Copb2,Tmed2,Tmed3,Tubb2a,Rab33b,Arfgap3,Rab3gap2,Pla2g4a,Tubb2b,Klc1,Gcc1,Cyth2,Copg1
MMU-1474290	Collagen formation	20	96	0.69	2.25e-06	Col1a1,Ctsb,P4ha1,Ctss,P4ha2,Plod1,Leprel2,P4hb,Col5a1,Pcolce,Ppib,Colgalt1,Crtap,Col5a2,Col3a1,Lepre1,Col14a1,Plod2,Serpinh1,Lox
MMU-948021	Transport to the Golgi and subsequent modification	20	177	0.43	0.0045	Tubb6,Ctsz,Cog1,Lman2,Arf4,Bet1l,Ctsc,Copb1,Arcn1,Copb2,Sec23ip,Tmed2,Tmed3,Tubb2a,Arfgap3,Tubb2b,Man2a1,Sec31a,Copg1,Mcfd2
MMU-72649	Translation initiation complex formation	19	57	0.9	8.64e-09	Pabpc1,Rps11,Rps9,Rps18,Rps26,Rps4x,Rps27l,Rps23,Rps12,Rps7,Rps25,Rps8,Rps6,Rps16,Rps19,Rps28,Rps15a,Rps20,Rps24
MMU-199977	ER to Golgi Anterograde Transport	19	147	0.49	0.0015	Tubb6,Ctsz,Cog1,Lman2,Arf4,Bet1l,Ctsc,Copb1,Arcn1,Copb2,Sec23ip,Tmed2,Tmed3,Tubb2a,Arfgap3,Tubb2b,Sec31a,Copg1,Mcfd2
MMU-69242	S Phase	19	147	0.49	0.0015	Mcm7,Pola1,Psme3,Mnat1,Mcm4,Rfc4,Fen1,Cdk2,Mcm6,Anapc2,Pcna,Anapc4,Mcm3,Mcm2,Cdc27,Cdc23,Mcm5,Cdkn1c,Psmb9

**MMU-72695**	Formation of the ternary complex, and subsequently, the 43S complex	18	50	0.93	9.12e-09	Rps11,Rps9,Rps18,Rps26,Rps4x,Rps27l,Rps23,Rps12,Rps7,Rps25,Rps8,Rps6,Rps16,Rps19,Rps28,Rps15a,Rps20,Rps24
**MMU-72702**	Ribosomal scanning and start codon recognition	18	57	0.87	4.85e-08	Rps11,Rps9,Rps18,Rps26,Rps4x,Rps27l,Rps23,Rps12,Rps7,Rps25,Rps8,Rps6,Rps16,Rps19,Rps28,Rps15a,Rps20,Rps24
**MMU-381426**	Regulation of Insulin-like Growth Factor (IGF) transport and uptake by Insulin-like Growth Factor Binding Proteins (IGFBPs)	18	122	0.54	0.000058	Rcn1,Hsp90b1,Matn3,P4hb,Chgb,Nucb1,Ckap4,Pdia6,Fn1,Lgals1,Golm1,Igf2,Tnc,Vcan,Spp1,Fstl1,Prkcsh,Apoe
**MMU-1650814**	Collagen biosynthesis and modifying enzymes	17	63	0.81	9.96e-07	Col1a1,P4ha1,P4ha2,Plod1,Leprel2,P4hb,Col5a1,Pcolce,Ppib,Colgalt1,Crtap,Col5a2,Col3a1,Lepre1,Col14a1,Plod2,Serpinh1
**MMU-2029480**	Fcgamma receptor (FCGR) dependent phagocytosis	17	82	0.69	2.30e-05	Vav1,Arpc2,Myh9,Fgr,Pak1,Nckap1l,Plcg2,Wasf2,Arpc1b,Arpc3,Pld3,Syk,Arpc4,Myo5a,Abi1,Myo9b,Actr3
**MMU-397014**	Muscle contraction	17	171	0.37	0.03557	Tpm4,Actn3,Myl4,Myh8,Myl6b,Vim,Pak1,Atp1b3,Itpr2,Itgb5,Myh6,Nos1,Camk2d,Myh3,Tnnt2,Dmd,Mybpc2

**MMU-8957275**	Post-translational protein phosphorylation	17	116	0.54	0.00098	Rcn1,Hsp90b1,Matn3,P4hb,Chgb,Nucb1,Ckap4,Pdia6,Fn1,Lgals1,Golm1,Tnc,Vcan,Spp1,Fstl1,Prkcsh,Apoe
**MMU-69239**	Synthesis of DNA	17	118	0.53	0.0012	Mcm7,Pola1,Psme3,Mcm4,Rfc4,Fen1,Cdk2,Mcm6,Anapc2,Pcna,Anapc4,Mcm3,Mcm2,Cdc27,Cdc23,Mcm5,Psmb9
**MMU-202733**	Cell surface interactions at the vascular wall	16	114	0.52	0.0023	Itgb2,Col1a1,Cd44,Itga5,Grb14,Atp1b3,Fn1,Itgam,Cd74,Ceacam1,Sirpa,Ppia,Cd84,Ptpn6,Slc3a2,Glg1
**MMU-8856688**	Golgi-to-ER retrograde transport	16	128	0.47	0.0066	Galnt1,Tubb6,Pafah1b3,Arf4,Copb1,Arcn1,Copb2,Tmed2,Tmed3,Tubb2a,Arfgap3,Rab3gap2,Pla2g4a,Tubb2b,Klc1,Copg1
**MMU-114608**	Platelet degranulation	15	122	0.46	0.0109	Itih3,Sparc,Habp4,Pcyox1l,Islr,Lamp2,Fn1,Lhfpl2,Cd109,Plek,Cd63,Psap,Igf2,Tagln2,Stxbp2
**MMU-2132295**	MHC class II antigen presentation	15	138	0.41	0.0294	Tubb6,Ctsb,Ctss,Lgmn,Ctsc,Ifi30,Tubb2a,H2-Eb1,Tubb2b,Klc1,Sec31a,Cd74,Clta,Ap1s1,Ctsd

**MMU-1474228**	Degradation of the extracellular matrix	15	144	0.39	0.00405	Col1a1,Cd44,Ctsb,Htra1,Ctss,Timp2,Col5a1,Acan,Mmp2,Fn1,Col5a2,Capn6,Col3a1,Spp1,Ctsd
**MMU-3000178**	ECM proteoglycans	14	50	0.82	1.12e-05	Col1a1,Vtn,Sparc,Matn3,Col5a1,Acan,Fn1,Itgb5,Col5a2,Col3a1,Matn1,Tnc,Vcan,Dag1
**MMU-2029482**	Regulation of actin dynamics for phagocytic cup formation	14	61	0.73	7.65e-05	Vav1,Arpc2,Myh9,Pak1,Nckap1l,Wasf2,Arpc1b,Arpc3,Syk,Arpc4,Myo5a,Abi1,Myo9b,Actr3
**MMU-216083**	Integrin cell surface interactions	14	79	0.62	0.000082	Itgb2,Col1a1,Cd44,Vtn,Itga5,Col5a1,Vcam1,Fn1,Itgam,Itgb5,Col5a2,Col3a1,Tnc,Spp1
**MMU-453279**	Mitotic G1 phase and G1/S transition	14	131	0.4	0.0443	Mcm7,Pola1,Psme3,Mnat1,Mcm4,Cdk2,Mcm6,Mcm3,Mcm2,Hdac1,Rbbp4,Mcm5,Cdkn1c,Psmb9
**MMU-73856**	RNA Polymerase II Transcription Termination	13	63	0.69	0.00044	Ddx39,Snrpf,Pabpn1,Cstf3,Ncbp1,Srsf9,Cstf2,Thoc2,Cpsf2,Papola,Srsf5,Srsf6,Srrm1

**MMU-69052**	Switching of origins to a post-replicative state	13	90	0.53	0.0070	Mcm7,Psme3,Mcm4,Cdk2,Mcm6,Anapc2,Anapc4,Mcm3,Mcm2,Cdc27,Cdc23,Mcm5,Psmb9
**MMU-6807878**	COPI-mediated anterograde transport	13	95	0.51	0.0106	Tubb6,Cog1,Arf4,Bet1l,Copb1,Arcn1,Copb2,Tmed2,Tmed3,Tubb2a,Arfgap3,Tubb2b,Copg1
**MMU-5357801**	Programmed Cell Death	13	116	0.42	0.0433	Ywhah,Apaf1,Ripk3,Casp8,Vim,Mlkl,Hist1h1a,Hmgb2,Bak1,Hist1h1b,Casp3,Dsp,Ripk1
**MMU-983231**	Factors involved in megakaryocyte development and platelet production	13	118	0.42	0.0483	Tubb6,Hdac2,Dock8,Tubb2a,Tubb2b,Dock10,Klc1,Dock2,Hdac1,Akap10,Kdm1a,Cbx5,Jmjd1c
**MMU-72187**	mRNA 3-end processing	12	54	0.72	0.00048	Ddx39,Pabpn1,Cstf3,Ncbp1,Srsf9,Cstf2,Thoc2,Cpsf2,Papola,Srsf5,Srsf6,Srrm1
**MMU-1236975**	Antigen processing-Cross presentation	12	92	0.49	0.0227	Calr,Cybb,Ncf1,Cyba,Psme3,Tapbp,Pdia3,Itgb5,Ncf4,Mrc2,Psmb9,Ncf2

**MMU-6811434**	COPI-dependent Golgi-to-ER retrograde traffic	12	94	0.48	0.02661	Tubb6,Arf4,Copb1,Arcn1,Copb2,Tmed2,Tmed3,Tubb2a,Arfgap3,Tubb2b,Klc1,Copg1
**MMU-2559583**	Cellular Senescence	12	124	1.8	4.79e-16	Rbbp4,Asf1a,Hist1h2af,Hist1h1a,Hist1h1b,Ehmt2,Cdk2,Anapc2,Anapc4,Cdkn1c,Cdc27,Cdc23
**MMU-05414**	Dilated cardiomyopathy	12	90	2.29	7.29e-24	Dmd,Sgcb,Sgcd,Sgcg,Dag1,Itga5,Itgb5,Cacng1,Adcy3,Tpm4,Tnnt2,Myh6
**MMU-05412**	Arrhythmogenic right ventricular cardiomyopathy	11	76	2.33	4.16e-22	Dsp,Ctnna3,Actn3,Dmd,Sgcb,Sgcd,Sgcg,Dag1,Itga5,Itgb5,Cacng1
**MMU-390522**	Striated Muscle Contraction	10	35	0.83	0.000044	Tpm4,Actn3,Myl4,Myh8,Vim,Myh6,Myh3,Tnnt2,Dmd,Mybpc2
**MMU-122**	ROS and RNS production in phagocytes	9	36	0.77	0.00022	Cybb,Ncf1,Cyba,Atp6v1e1,Atp6v1c1,Ncf4,Nos1,Atp6v1a,Ncf2

**2556**						
**MMU-68962**	Activation of the pre-replicative complex	8	32	0.77	0.0047	Mcm7,Pola1,Mcm4,Cdk2,Mcm6,Mcm3,Mcm2,Mcm5
**MMU-3299685**	Detoxification of Reactive Oxygen Species	8	34	0.75	0.0066	Cybb,Ncf1,Cyba,Gpx8,P4hb,Gpx7,Ncf4,Ncf2
**MMU-5663213**	RHO GTPases Activate WASPs and WAVEs	8	34	0.75	0.0066	Arpc2,Nckap1l,Wasf2,Arpc1b,Arpc3,Arpc4,Abi1,Actr3
**MMU-176187**	Activation of ATR in response to replication stress	8	37	0.71	0.0101	Mcm7,Mcm4,Rfc4,Cdk2,Mcm6,Mcm3,Mcm2,Mcm5
**MMU-75067**	Processing of Capped Intronless Pre-mRNA	7	28	0.77	0.0106	Snrpf,Pabpn1,Cstf3,Ncbp1,Cstf2,Cpsf2,Papola
**MMU-8964043**	Plasma lipoprotein clearance	7	30	0.74	0.0147	Npc2,Apobr,Lipa,Clta,Hdlbp,Apoe,Soat1

**MMU-2173782**	Binding and Uptake of Ligands by Scavenger Receptors	7	35	0.67	0.02889	Calr,Sparc,Hsp90b1,Msr1,Stab1,Hp,Apoe
**MMU-6791312**	TP53 Regulates Transcription of Cell Cycle Genes	7	35	0.67	0.02889	Cdk2,Pcna,Cnot3,Npm1,Cnot2,Cnot8,Cdkn1c
**MMU-3000171**	Non-integrin membrane-ECM interactions	7	37	0.65	0.03669	Col1a1,Vtn,Col5a1,Fn1,Itgb5,Col5a2,Col3a1
**MMU-2559582**	Senescence-Associated Secretory Phenotype (SASP)	7	40	0.62	0.04890	Ehmt2,Cdk2,Anapc2,Anapc4,Cdc27,Cdc23,Cdkn1c
**MMU-1236973**	Cross-presentation of particulate exogenous antigens (phagosomes)	6	8	1.01	0.00044	Cybb,Ncf1,Cyba,Itgb5,Ncf4,Ncf2
**MMU-77595**	Processing of Intronless Pre-mRNAs	6	19	0.87	0.01011	Pabpn1,Cstf3,Ncbp1,Cstf2,Cpsf2,Papola

**MMU-8956321**	Nucleotide salvage	6	23	0.79	0.02077	Gmpr2,Ada,Upp1,Hprt,Uck2,Ampd2
**MMU-5668599**	RHO GTPases Activate NADPH Oxidases	6	24	0.77	0.02443	Cybb,Ncf1,Cyba,Ncf4,S100a9,Ncf2
**MMU-176408**	Regulation of APC/C activators between G1/S and early anaphase	6	29	0.69	0.04883	Cdk2,Anapc2,Anapc4,Cdc27,Mad2l1,Cdc23
**MMU-3000480**	Scavenging by Class A Receptors	4	10	0.98	0.03888	Calr,Hsp90b1,Msr1,Apoe
**MMU-04260**	Cardiac muscle contraction	4	84	1.85	1.73e-05	Myh6,Tnnt2,Tpm4,Cacng1
**MMU-71288**	Creatine metabolism	4	10	0.98	0.03888	Ckmt1,Ckb,Gatm,Gamt

**MMU-1566977**	Fibronectin matrix formation	3	4	1.01	0.04002	Itga5,Fn1,Ceacam1
