# Supplementary figures and images for: Proteome Profiling of the Dystrophic mdx Mice Diaphragm
Source: Biomolecules. 2023 Nov 13;13(11):1648. doi: 10.3390/biom13111648 (PMC10669179; doi:10.3390/biom13111648)

## Slide 1
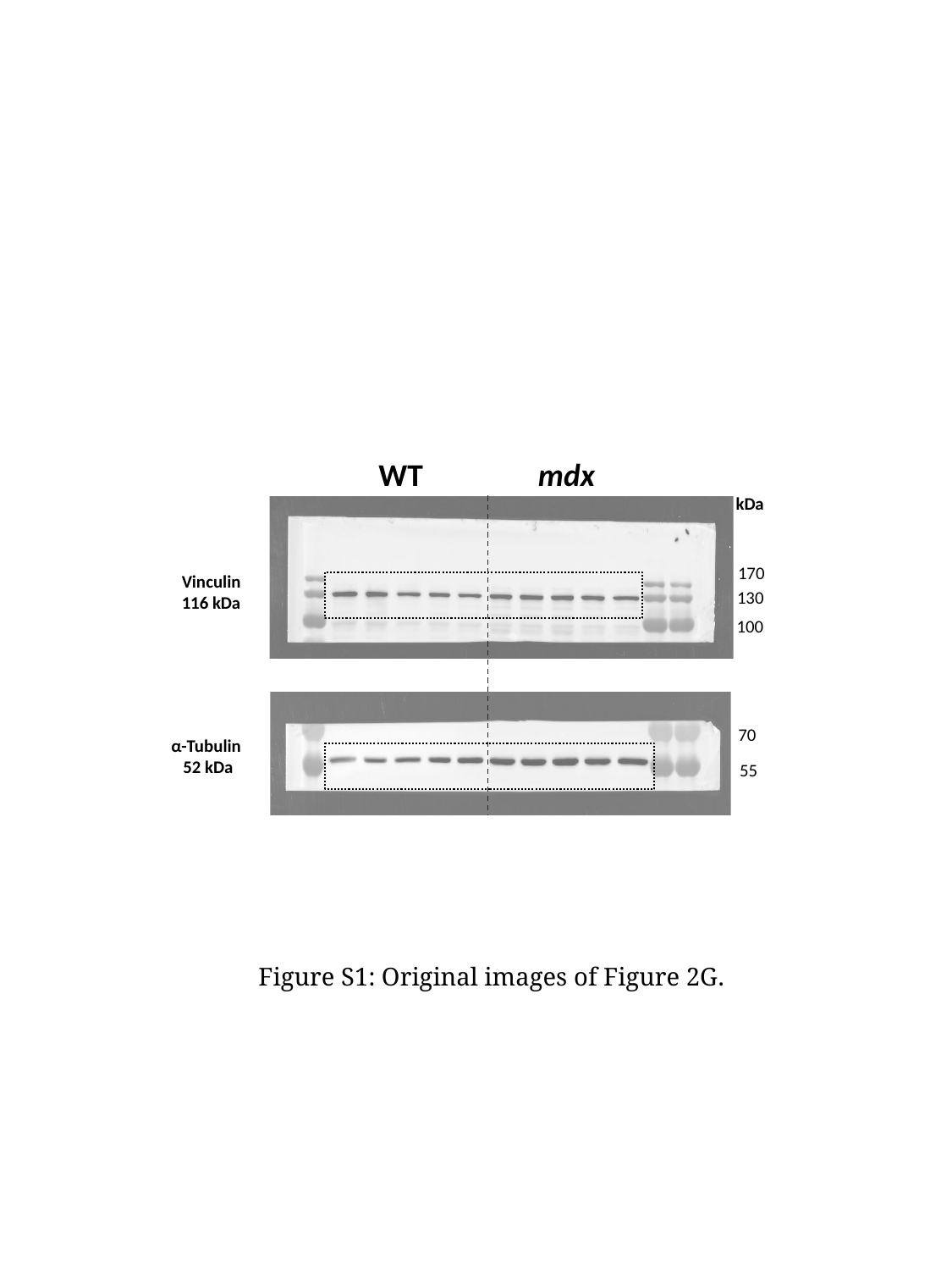

WT mdx
kDa
170
Vinculin
116 kDa
130
100
70
α-Tubulin
52 kDa
55
Figure S1: Original images of Figure 2G.

Supplement: Supplementary file 1 [file biomolecules-13-01648-s001.zip › Figure S1.pptx]

## Slide 1
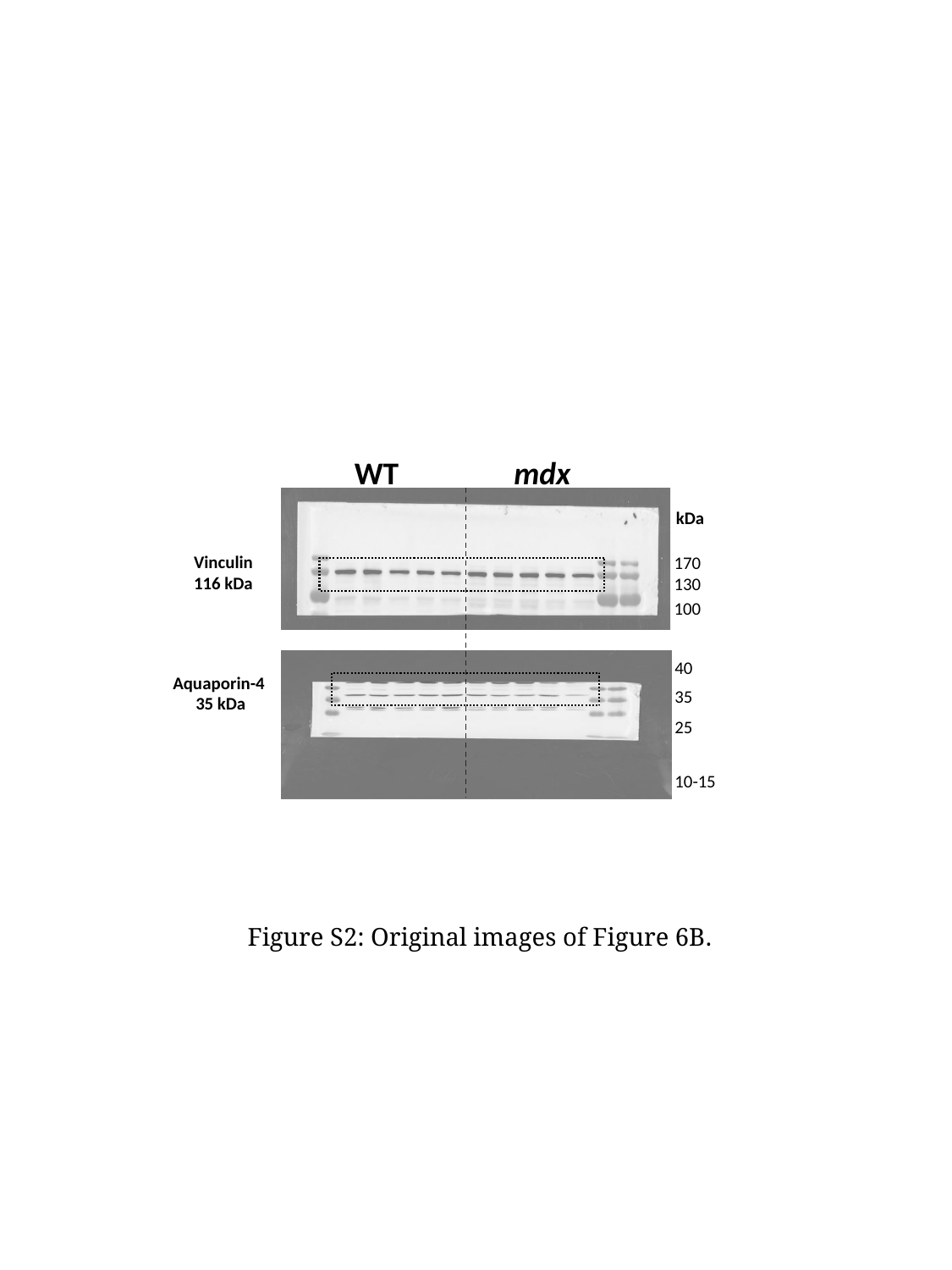

WT mdx
kDa
Vinculin
116 kDa
170
130
100
40
Aquaporin-4
35 kDa
35
25
10-15
Figure S2: Original images of Figure 6B.

Supplement: Supplementary file 1 [file biomolecules-13-01648-s001.zip › Figure S2.pptx]

## Slide 1
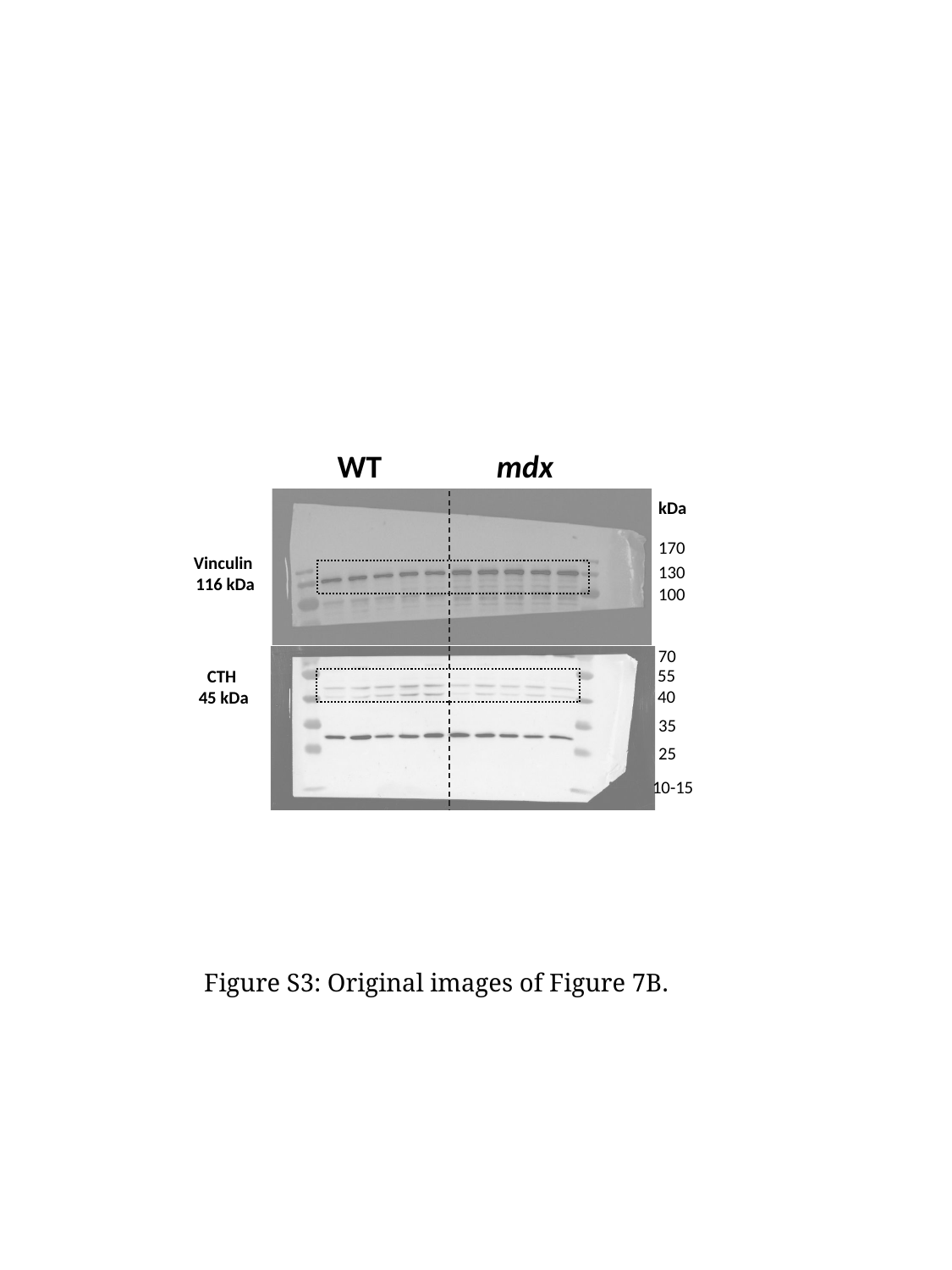

WT mdx
kDa
170
Vinculin
116 kDa
130
100
70
55
CTH
45 kDa
40
35
25
10-15
Figure S3: Original images of Figure 7B.

Supplement: Supplementary file 1 [file biomolecules-13-01648-s001.zip › Figure S3.pptx]

## Slide 1
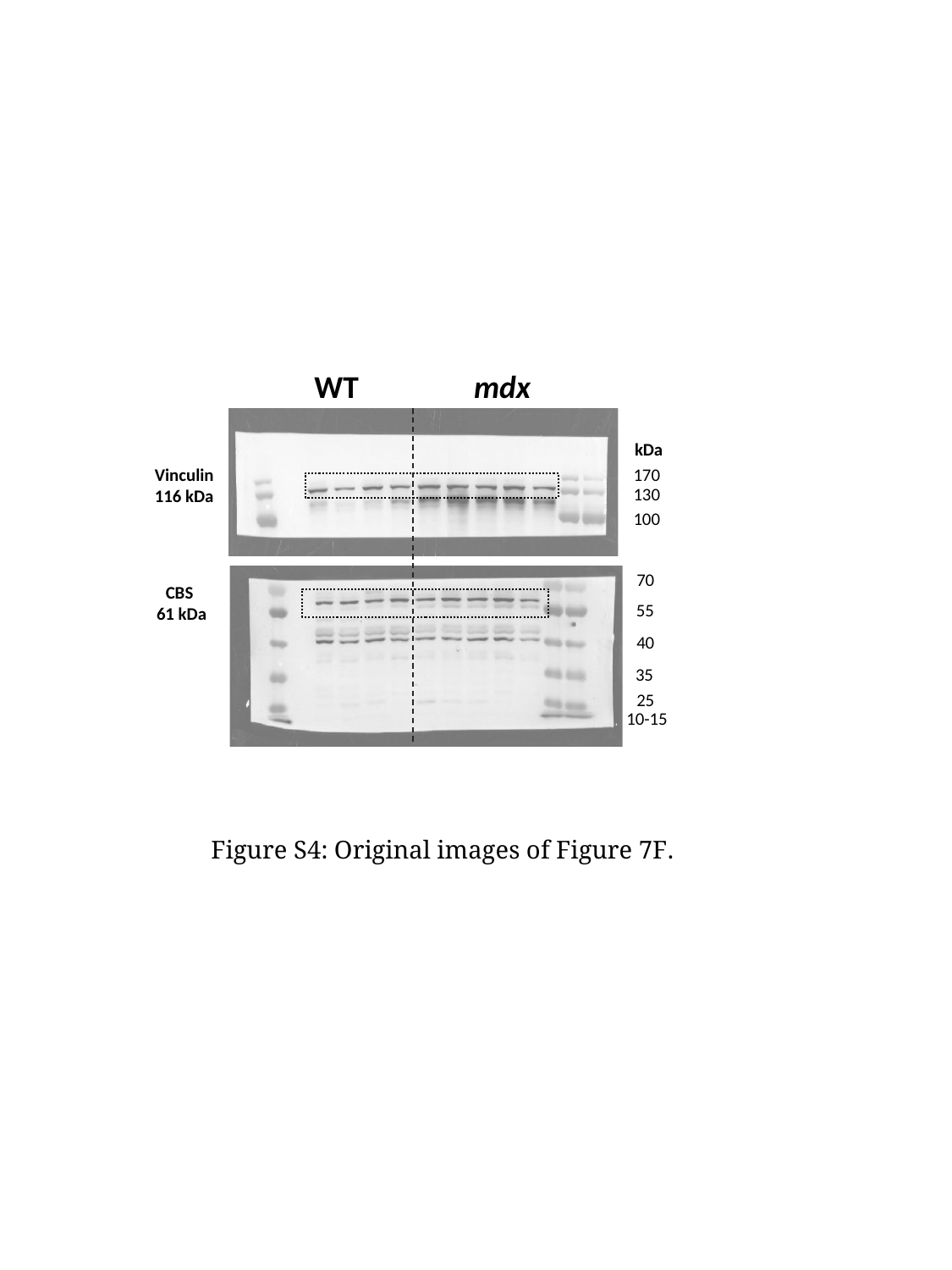

WT mdx
kDa
Vinculin
116 kDa
170
130
100
70
CBS
61 kDa
55
40
35
25
10-15
Figure S4: Original images of Figure 7F.

Supplement: Supplementary file 1 [file biomolecules-13-01648-s001.zip › Figure S4.pptx]

## Slide 1
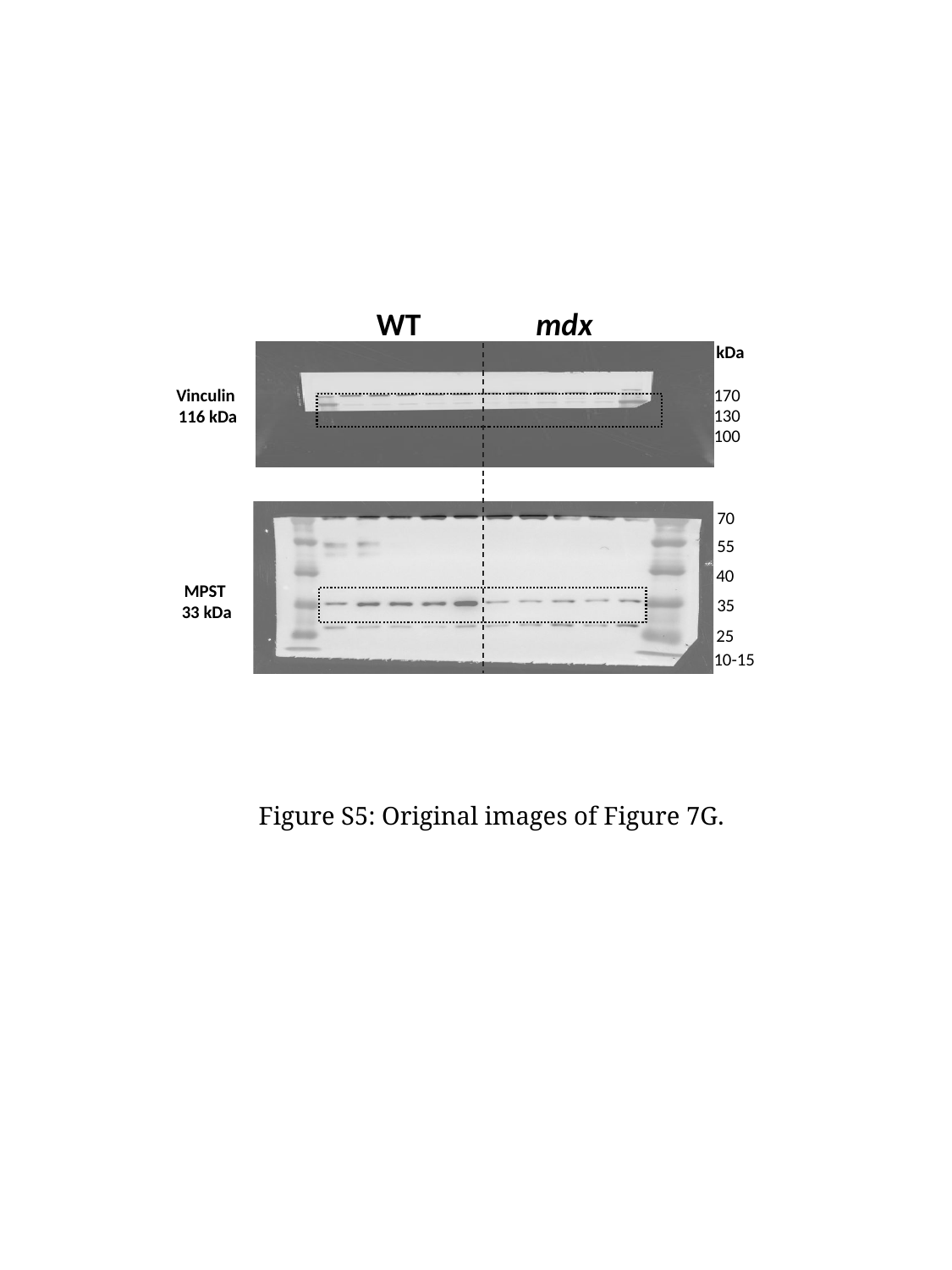

WT mdx
kDa
Vinculin
116 kDa
170
130
100
70
55
40
MPST
33 kDa
35
25
10-15
Figure S5: Original images of Figure 7G.

Supplement: Supplementary file 1 [file biomolecules-13-01648-s001.zip › Figure S5.pptx]
